# The microbiota–gut–brain axis as a modulator of symptom expression in autism spectrum disorder, with exploratory insights into ADHD: evidence from a structured narrative review on paediatric population

**DOI:** 10.3389/frcha.2026.1835043

**Published:** 2026-05-28

**Authors:** Alessandra Carta, Maria Pia Riccio, Alessandro Miola, Vanna Cavassa, Mariangela Valentina Puci, Giuseppe Abbracciavento, Sara Carucci, Fabio Sambataro, Carmela Bravaccio, Stefano Sotgiu

**Affiliations:** 1 Division of Child Neuropsychiatry, Department of Medicine, Surgery and Pharmacy, University of Sassari, Sassari, Italy; 2 Department of Maternal and Child Health, Child and Adolescent Psychiatry, Azienda Ospedaliera Universitaria Federico II, Naples, Italy; 3 Department of Neuroscience, University of Padova, Padua, Italy; 4 Department of Psychiatry & Psychology, Mayo Clinic, Rochester, MN, United States; 5 Department of Medicine and Surgery, Kore University of Enna, Enna, Italy; 6 Child and Adolescent Neuropsychiatry Unit, Pediatric Hospital “A.Cao” Microcitemico, Cagliari, Italy; 7 Department of Medical Science and Public Health, University of Cagliari, Monserrato, Italy; 8 Padova Neuroscience Center, University of Padova, Padua, Italy; 9 Child Neuropsychiatry, Department of Medical and Translational Sciences, Federico II University, Naples, Italy

**Keywords:** ADHD, autism spectrum disorder, diet, gut–brain axis, microbiota, review

## Abstract

Autism spectrum disorder (ASD) and attention-deficit/hyperactivity disorder (ADHD) are increasingly conceptualized as multisystem neurodevelopmental conditions involving behavioral, gastrointestinal, immune, and metabolic alterations. The microbiota–gut–brain axis (MGBA) has been proposed as a key framework linking these systems, although evidence from human studies remains heterogeneous and inconclusive. We conducted a structured narrative review of pediatric human studies investigating the microbiota-gut-brain axis (MGBA) in ASD and ADHD, excluding preclinical and animal studies. PRISMA 2020 principles were used as a transparency framework to support reporting of study identification, screening, and eligibility. Observational, cohort, and interventional studies were synthesized with particular attention to study design, methodology, associations with standardized clinical scales, dietary factors, and therapeutic implications. Across ASD (*n* = 90), most studies were observational, predominantly cross-sectional or case-control in designs, reporting consistent associations between ASD symptom severity and gastrointestinal symptoms, intestinal permeability, immune activation, and dietary selectivity. Evidence in ADHD (*n* = 21) was substantially more limited and largely observational. Diet emerged as a major modulating and confounding factor in microbiota–behavior associations. Interventional studies involving probiotics, microbiota transfer therapy, and other gut-directed approaches showed more consistent improved gastrointestinal outcomes but showed modest and heterogeneous effects on core ASD symptoms, often limited to subgroups with prominent gastrointestinal comorbidities. To strengthen interpretation of interventional findings, randomized trial reports and non-randomized interventional studies were appraised according to study design, whereas the predominantly heterogeneous observational literature was interpreted narratively. Overall, this review suggests that MGBA alterations may be better conceptualized as modifiers of symptom expression, particularly in ASD and with only emerging relevance in ADHD, rather than as disorder-specific causal mechanisms. This interpretation underscores the need for stratified, longitudinal, and clinically informed research frameworks, in line with the focus of the present research topic.

## Introduction

Autism spectrum disorder (ASD) and attention-deficit/hyperactivity disorder (ADHD) are two clinically relevant neurodevelopmental disorders with partially overlapping features that may be particularly informative for microbiota-gut-brain-axis (MGBA) research. ASD and ADHD share several transdiagnostic dimensions potentially relevant to gut-brain mechanisms, including behavioral and emotional dysregulation and altered stress responsivity (1–3). In addition to core behavioral symptoms, both ASD and ADHD are frequently associated with gastrointestinal disturbances, immune dysregulation, metabolic alterations, and atypical dietary patterns, suggesting the involvement of systemic biological pathways interacting with neurodevelopment (4, 5). Moreover, both conditions have been associated with alterations in neurotransmitter systems, particularly serotonergic and dopaminergic pathways, which may be influenced by gut microbial signalling through neural, endocrine, immune, and metabolic routes (6, 7). Within this framework, ASD and ADHD can be viewed as complementary neurodevelopmental models through which to explore whether MGBA-related alterations primarily modulate symptom expression across neurodevelopmental phenotypes, rather than representing disorder-specific causal signatures.

Over the past decade, the microbiota–gut–brain axis (MGBA) has emerged as a key conceptual framework linking intestinal microbial communities with neural development, immune signaling, and behavior through bidirectional communication pathways involving neural, endocrine, and immune mechanisms (8, 9). Human studies in ASD have reported alterations in gut microbiota composition compared with neurotypical controls; however, findings remain heterogeneous and no reproducible, ASD-specific microbial signature has been consistently identified across cohorts (10, 11).

Importantly, gastrointestinal symptoms and dietary selectivity are highly prevalent in children with ASD and may strongly influence observed microbiota profiles, raising questions about the directionality and clinical significance of microbiota–behavior associations (12, 13). Similar but far more limited evidence exists for ADHD, where available studies suggest potential MGBA involvement, but they are characterized by small sample sizes and predominantly observational designs (14, 15).

Beyond postnatal factors, emerging evidence implicates immune mechanisms, including maternal immune activation and immune dysregulation in affected children, as contributors to altered neurodevelopmental trajectories that may interact with gut microbiota composition and function (16, 17). Collectively, these observations support the view that MGBA-related alterations may act as modulatory factors influencing symptom expression, rather than as primary disorder-specific causal mechanisms.

Based on this rationale, we conducted a structured narrative review of the pediatric human literature on the microbiota–gut–brain axis in ASD and ADHD. The primary aim of the present structured narrative review is to explore clinically relevant pediatric human studies investigating the microbiota–gut–brain axis in ASD and ADHD. Specifically, this review aims to: (i) describe the design of available study; (ii) summarize the available evidence on the associations between microbiota-related findings, gastrointestinal symptoms, dietary patterns, and standardized clinical scales; and (iii) discuss the potential therapeutic implications of microbiota- and diet-targeted interventions in relation to both gastrointestinal outcomes, core neurodevelopmental symptoms, and the available evidence on these associations.

## Methods

### Search strategy and study selection criteria

This manuscript was designed as a structured narrative review, and PRISMA 2020 principles were used solely to enhance transparency in the reporting study identification and selection, without implying a formal systematic review methodology (18). The literature search was designed to identify clinically relevant pediatric human studies investigating the MGBA in individuals diagnosed with ASD and ADHD, in order to support a focused and transparent narrative synthesis. Studies published between 2015 and 2025 were considered in order to capture the most recent decade of research, reflecting the rapid expansion of microbiome–gut–brain axis studies following the widespread adoption of next-generation sequencing technologies and the consolidation of diagnostic criteria after the introduction of DSM-5. Relevant studies were identified through searches in PubMed/MEDLINE, Web of Science, and PsycINFO using combinations of free-text terms and Medical Subject Headings (MeSH), including, “autism spectrum disorder,” “ASD,” “attention-deficit/hyperactivity disorder,” “ADHD,” “gut microbiota,” “microbiome,” “microbiota–gut–brain axis,” “gastrointestinal symptoms,” and “diet.”

Studies were included if they: (i) involved human participants aged 0–18 years with a diagnosis of ASD or ADHD; (ii) investigated gut microbiota composition, MGBA-related mechanisms, and/or microbiota-targeted interventions; and (iii) were original research articles employing observational or interventional designs, including cross-sectional, case–control, and cohort study,, as well as open-label or randomised control trials (RCTs).

Preclinical and animal studies were intentionally excluded because the primary aim of this review was rather to synthesize clinically relevant human evidence on how MGBA-related alterations may be associated with symptom expression and treatment response in pediatric population with ASD and ADHD conditions. Given the substantial translational gap between preclinical findings and clinically observable neurodevelopmental phenotypes, restricting the evidence base to pediatric human studies was considered necessary to preserve the clinical applicability and interpretive consistency of the review.

To ensure clinical relevance and consistency of interpretation, adult-only samples and non-English publications were explicitly excluded. Narrative reviews, systematic reviews, and meta-analyses, along with conference abstracts, editorials, commentaries, letters to the editor, case reports, and studies lacking original data, were excluded to maintain the focus on primary pediatric human data.

Since this paper is a narrative review rather than a systematic one, the final selection of articles was guided by the authors’ qualitative judgment of relevance and methodological robustness of the studies. Given the narrative nature of this review and its aim to provide a qualitative and conceptually driven synthesis of the literature, a formal risk of bias or quality assessment of individual studies was not performed (130).

## Results

**Figure 1:**
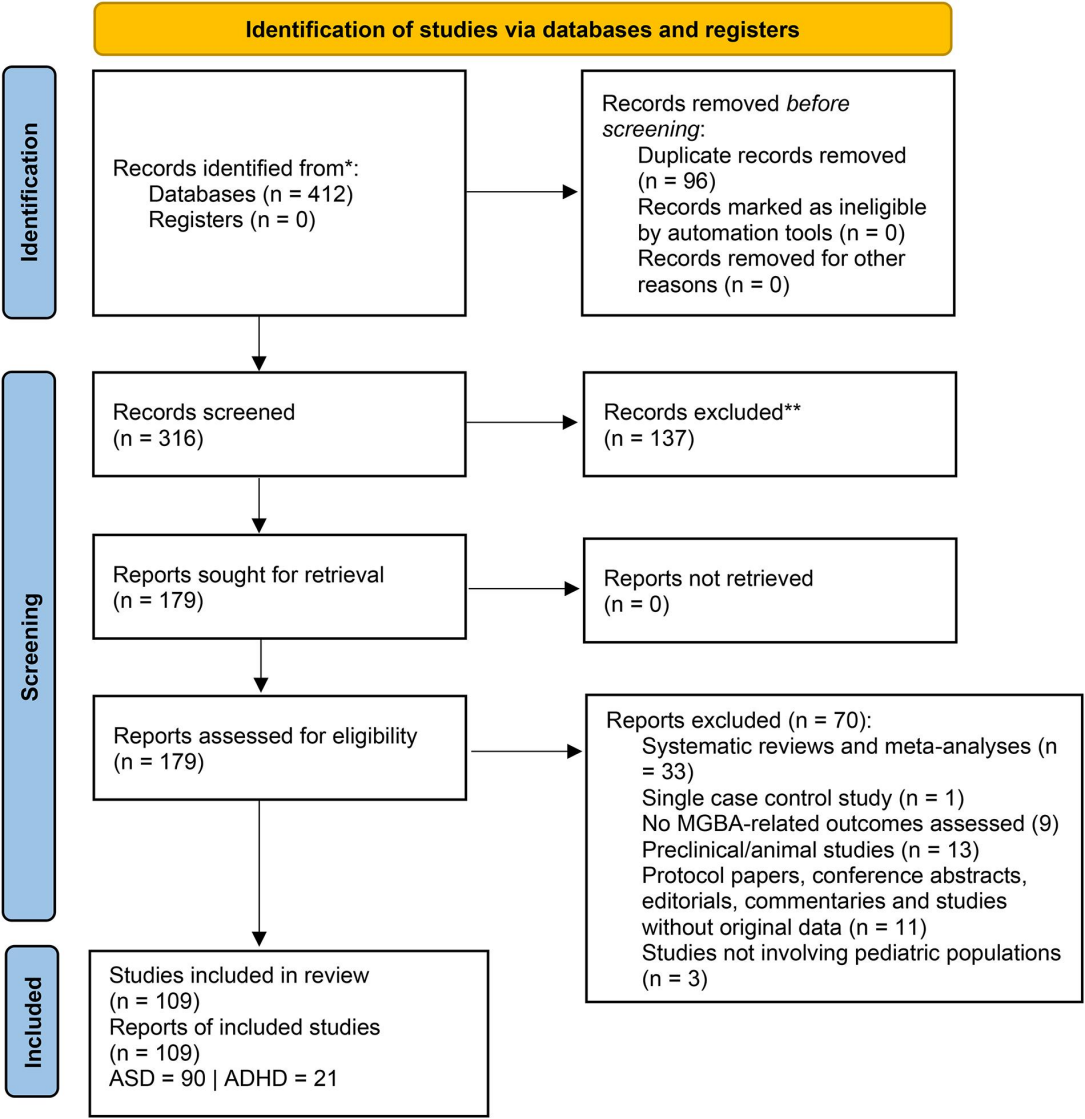
PRISMA 2020 flow diagram of the study selection process. The diagram illustrates the selection workflow from the initial search to the final inclusion. A total of 412 initial records were identified from databases (2015–2025). After the removal of 96 duplicates, 316 articles were screened, leading to the exclusion of 137 records due to a lack of relevance (e.g., conditions other than ASD or ADHD, lack of original data, non-pediatric populations). Of the 179 articles assessed in full text, 70 were excluded for specific reasons; systematic reviews/meta-analyses (*n* = 33), preclinical/animal studies (*n* = 13), protocol papers or editorials (*n* = 11), absence of outcomes related to the microbiodata-gut-brain axis (*n* = 9), non-pediatric studies (*n* = 3), and a single case study (*n* = 1). A total of 109 studies were included in the final review (90 for ASD and 21 for ADHD) *From*: Page MJ, McKenzie JE, Bossuyt PM, Boutron I, Hoffman TC, Mulrow CD, et al. The PRISMA 2020 statement: an updated guideline for reporting systematic reviews. BMJ 2021; 372:n71. doi: 10.1136/bmj.n71 For more information, visit http://www.prisma-statement.org/.

A total of 412 records published between 2015 and 2025 were identified through the databases search. After removal of 96 duplicate records, 316 articles remained and were screened at the title and abstract level. During this phase, records were excluded at the title and abstract screening stage (*n* = 137) for the following reasons: studies addressing neurological or psychiatric conditions other than ASD or ADHD; articles focusing on peripheral microbiota without reference to gut–brain interactions; studies not involving pediatric populations; and publications that were reviews, commentaries, opinion pieces, or conference abstracts lacking original data. Full texts of 179 articles were subsequently assessed for eligibility. Of these, 70 studies were excluded for the following reasons: (i) systematic review and meta-analyses (*n* = 33), to avoid the risk of duplication data; (i) single case report (1); (iii) studies not reporting a clear outcome on gut-brain axis (*n* = 9); (iv) preclinical or animal studies (*n* = 13); (v) protocol papers, conference abstracts, editorials, commentaries and studies without original data (*n* = 11); and (vi) studies not involving pediatric populations (*n* = 3), in line with the predefined inclusion criteria. Following full-text screening, 109 studies met all inclusion criteria and were included in the qualitative synthesis (Figure 1).

### Study characteristics and distribution by diagnostic group

Among the included studies, 90 focused on individuals with ASD and 21 on individual with ADHD. Two studies were counted in both the ASD and ADHD groups because they included mixed clinical samples comprising participants with both diagnoses and therefore contributed data relevant to both diagnostic categories.

Supplementary Table 1 (4, 5, 11–13, 19–103) and Supplementary Table 2 (14, 15, 63, 77, 104–120) summarize the characteristics of the ASD and ADHD studies, respectively. Across both diagnostic groups, the evidence base was predominantly observational, with most studies adopting cross-sectional or case-control designs, whereas interventional studies represented a substantially smaller and methodologically more heterogeneous subset. The temporal distribution of publications showed a clear increase in the number of ASD-related studies over the last decade, whereas ADHD-focused investigations remained comparatively sparse throughout the entire study period.

**Table 1:** Characteristics of pediatric human studies investigating microbiota–gut–brain axis-related mechanisms, gastrointestinal features, dietary factors, and intervention outcomes in attention-deficit/hyperactivity disorder.

Citation (Author, year)	Reference	Study design	Age range	Sample size	DSM and/or 1CD diagnostical features	Methodological approaches across studies	Behavioural scale	Clinical associations 1nvolving ADHD symptoms, gastrointestinal features, and diet	Interventional studies and clinical outcomes	Main findings
Pärtty et al. (2015) 1038/pr.2015.51	(104)	RCT - secondary analysis	Birth–13	75 (probiotic 40; placebo 35)	ICD10	Randomized early-life probiotic supplementation (Lactobacillus rhamnosus GG); long-term follow-up; psychiatric diagnosis 6a 1CD-10	NR	Early probiotic exposure–ADHD risk: ↓; GI features: NA; Diet: NA	Probiotic supplementation 1n 1nfancy; outcome: later diagnosis of ADHD or ASD	Early-life probiotic supplementation associated with lower risk of later ADHD diagnosis
Özyurt et al. (2018)	(105)	C-CS	7–12	81 (ASD 40; CG 41)	DSM-5	serum zonulin (ELISA); DuPaul ADHD Rating Scale; SRS; K-SADS-PL; correlation and linear regression analyses	DuPaul ADHD-RS-IV; SRS; K-SADS-PL	Zonulin ↑ ADHD vs CG; Zonulin–ADHD SS (hyperactivity): ↑; Zonulin–SRS: ↑; GI features: NA; Diet: NA	NA	Higher serum zonulin levels in ADHD associated with greater hyperactivity and social dysfunction
Prehn-Kristensen et al. (2018)	(14)	C-CS	7–14	31 (ADHD 14; CG 17)	DSM-IV	FSC; 16S rRNA GS; BTA; alpha and beta diversity analyses; differential taxa analysis	CBCL; FBB-HKS; WURS-k; ADHS-SB	GMC alpha-diversity ↓ ADHD vs CG; GI features: NA; Diet: NA	NA	Reduced gut microbiome alpha diversity observed in children with ADHD compared with controls
Stevens et al. (2019)	(106)	RCT - secondary analysis	7–12	17 children (10 treatment/7 placebo)	NR	Micronutrient supplementation; FSC (longitudinal); 16S rRNA GS; BTA; GMC diversity analyses; ADHD clinical rating scales	ADHD-RS-IV; CGAS	Micronutrients–GMC: ≠ (longitudinal change); Micronutrients–ADHD SS: ↓; Diet quality–ADHD SS: Ø; GI features: NA	Micronutrient supplementation (10 weeks); GMC modulation during treatment	Micronutrient supplementation altered gut microbiome composition during treatment; ADHD symptom improvement was reported and appeared independent of overall diet quality.
Fernández-López et al. (2020)	(107)	Prospective controlled observational study with repeated measures	5–14	179 (ADHD 130; CG 49)	DSM-IV-TR	BSC (evening and morning); urine collection; LC–MS/MS for indole and tryptophan-derived metabolites; S100B measurement (ELISA); methylphenidate treatment; factorial ANOVA; subgroup analyses (PAD; PHI)	EDAH; CDI; d2 Test	Indole metabolites–ADHD SS: profile alteration; Indole metabolites–depressive symptoms (PHI): ↑ baseline, ↓ post-treatment; GI features: NA; Diet: NA	Methylphenidate (∼4.6 months); normalization of diurnal indole metabolite profiles; S100B diurnal profile modulation	ADHD associated with altered diurnal indole tryptophan metabolite profiles; methylphenidate partially restored control-like rhythms, especially in PHI with depressive symptoms
				*ADHD sample size differs between Methods and* Tables (107 vs 130)*.*						
Kumperscak et al. (2020)	(108)	pilot RCT not registered	4–17	32	DSM-5	Randomized allocation; probiotic supplementation (Lactobacillus rhamnosus GG); ADHD Rating Scale; Conners’ Parent Rating Scale; adverse event monitoring	ADHD-RS-IV; PedsQL 4.0; CBCL 6–18; TRF; ASEBA	ADHD SS post-LGG vs baseline: ↓; LGG vs placebo: Ø; GI features: NA; Diet: NA	Lactobacillus rhamnosus GG supplementation; no significant benefit vs placebo on ADHD core symptoms; good tolerability	LGG supplementation was safe but did not significantly improve ADHD symptoms compared with placebo
Pan et al. (2020)	(109)	CS-CO-TWIN	4–17	382	DSM-5	Parent-reported questionnaires; CBCL/ABCL Attention Problems subscale; structured physical health questionnaires; co-twin control analyses	CBCL;ABCL-AP; K-SADS-PL; DIVA 2.0; ASEBA	GI conditions ↑ ADHD vs non-ADHD co-twins; Physical health problems ↑ ADHD vs non-ADHD co-twins; Diet: NA	NA	ADHD associated with increased physical and gastrointestinal health problems after controlling for shared genetic and environmental factors
Jung TH et al. (2022)	(110)	C-CS	11–12	40 (ADHD 7; CG 33)	NR – ADHD defined by K-ARS (scale-based)	16S rRNA; qPCR; GC-MS (SCFA); BTA	K-ARS Rating Scale (ARS)-VI	Processed food diet ↑ ADHD score; ADHD group: ↓ α-diversity; ↑ Enterobacter, ↑ Escherichia; ↓ Bifidobacterium, ↓ Faecalibacterium, ↓ Ruminococcus; ↓ acetate, ↓ butyrate	NA	Processed food dietary pattern associated with GMC dysbiosis and ↓ SCFA; ADHD symptoms correlated with altered GMC and ↓ diversity.
Lee (2022)	(111)	C-CS	6–18	76 (ADHD 54; CG 22)	DSM-5	16S rRNA; OTU (97%); UniFrac; LEfSe; BTA; QIIME	SNAP-IV; CBCL	α-diversity Ø; β-diversity ≠ (UniFrac, *p* < 0.001); ADHD: ↑ Proteobacteria; ↑ Agathobacter; ↑ Ruminococcus_gnavus_group; CG: ↑ Alistipes; ↑ Rikenellaceae; GMC ↔ SNAP-IV Ø; Agathobacter ↑ ↔ Withdrawn/Depressed (CBCL); Ruminococcus_gnavus_group ↑ ↔ Externalizing (CBCL).	NA	ADHD associated with altered GMC (β-diversity ≠, α-diversity Ø). Specific genera (Agathobacter, Ruminococcus_gnavus_group) associated with emotional–behavioral symptoms independent of core ADHD severity.
Perham et al. (2022)	(112)	EXPLORATORY-CC	7–12	107 (55 ADHD; 52 CG)	NR	Hair sample collection; elemental analysis of metals and trace elements; clinical ADHD diagnostic assessment	ADHD-RS-IV; KSADS-PL	Hair elemental profiles ≠ ADHD vs CG; GI features: NA; Diet: NA	NA	Altered hair elemental balance observed in children with ADHD compared with controls
Yang et al. (2022)	(113)	C-CS	children: 5–17 years; adults up to 55 years	269 (ADHD 233; GC 36)	ICD10	ICD-10 ADHD diagnosis (F90); plasma SCFA quantification (LC–MS); antibiotic exposure history; ADHD medication data; BMI; serum vitamin D; dietary FFQ with PCA; correlation and multivariable regression analyses	NR	Plasma SCFA ↓ ADHD vs CG (adults); Plasma SCFA–antibiotic exposure: ↓; Plasma SCFA–ADHD SS: Ø; Plasma SCFA–dietary PC2 (acetic acid): ↑; GI features: Ø; Diet: assoc (PC-specific)	NA	People with ADHD showed reduced plasma SCFA levels, partly related to antibiotic exposure and dietary patterns, with no association with ADHD symptom severity.
Elhossiny et al. (2023)	(114)	RCT	6–16	80 (INT 40; CG 40); Aalyzed 76 (INT 36; CG 40)	DSM-5	Computer-based randomization; assessor-blinded; INT (probiotic + atomoxetine) vs CG (atomoxetine); baseline and follow-up	CPRS-R; CBCL 6–18	ADHD ↓ (INT vs CG); GI: NA; diet: NA; GMC: hypothesized (GBA rationale)	Probiotic: Lactobacillus acidophilus LB + atomoxetine → ADHD ↓; focused attention ↑ (CPT); EF ↑ partial (WCST); impulsivity: NSA	Significant ADHD ↓ (CPRS-R, CBCL); sustained attention ↑; EF ↑ partial; adverse events Ø relevant; GI NA
Kurokawa et al. (2024)	(63)	C-CS	6–12	n 98 (ASD 18; ADHD 19; ASD + ADHD 20; Siblings 13; Unrelated control 28)	DSM-5	FSC; 16S rRNA GS; BTA; GMC; alpha diversity (Chao1, Shannon, Faith's PD); dietary questionnaire; GSS; ADOS-2; CARS	Conners-3; SSP	Dietary diversity ↓ ADHD vs CG: ↑; GMC alpha-diversity ↓ ADHD vs CG: ↑; Dietary diversity–GMC: ↑; GI features (GSRS): NA; Diet–ADHD SS: NA	NA	Lower dietary diversity was associated with reduced gut microbial diversity in children with ADHD and other neurodevelopmental conditions
Petropoulos et al. (2024)	(77)	C-CS	4–12	n 120 (ASD 40; ADHD 40; CG 40)	DSM-5	Hair elemental analysis; salivary cortisol; functional GI symptom questionnaire; group comparisons	CBCL	FGIDs ↑ ADHD vs CG; Biological profiles (hair elements, cortisol) ≠ ADHD vs CG; Diet: NA	NA	ADHD associated with increased functional gastrointestinal symptom burden and distinct biological profiles compared with controls
Robinette et al. (2024)	(115)	RCT - secondary analysis	6–12	124 (multinutrients 69; placebo 55)	DSM-5	Randomized multinutrient supplementation; clinician-rated CGI-I; CGI-S; dietary assessment (Healthy Eating Index-2015); moderator analyses	CGI-S; CGI-I; CASI-5	Multinutrients–ADHD SS: ↓; Diet quality–ADHD SS: Ø; Vegetable intake–treatment response: ↑; GI features: NA	Broad-spectrum multinutrient supplementation (8 weeks); higher CGI-I/CGI-S improvement vs placebo	Multinutrient supplementation improved ADHD clinical outcomes independent of overall diet quality
Steckler (2024)	(117)	C-CS	6–18	73 (ADHD 42; CG 31)	DSM-5	16S rRNA; QIIME2; LEfSe; GC-MS (SCFA); BTA	NA	α-diversity ↓ (Shannon; Faith PD; Observed species); β-diversity NSA (trend Weighted UniFrac); ADHD: ↑ Blautia; ↑ Lachnospiraceae; CG: ↑ Verrucomicrobia; ↑ Akkermansia; ↑ Ruminococcaceae; SCFA: ↓ acetate; ↓ propionate; ↓ isobutyrate; ↓ isovalerate; ↓ valerate; butyrate NSA	NA	ADHD associated with GMC dysbiosis (↓ α-diversity) and ↓ SCFA levels, suggesting altered microbial–metabolic profile within MGBA.
Ast (2025)	(116)	RCT - secondary analysis	6–12	44	DSM-5	16S rRNA; QIIME2; ASV; LEfSe; BTA	CASI-5; CGI-S; CGI-I	Baseline GMC ≠ responders vs non-responders; Treatment-associated GMC shifts; No consistent baseline α-diversity differences"	Micronutrient treatment vs placebo (8 weeks); Clinical improvement associated with specific GMC signatures	Micronutrient treatment modulates GMC; baseline microbial profile associated with treatment response in ADHD.
Boonchooduang et al. (2025)	(15)	CS	6–12	25 ADHD (NO CG)	DSM-5	Clinical ADHD diagnosis; sleep questionnaires; FSC; fecal SCFA quantification; microbiota-derived metabolite analysis	SNAP-IV; CSHQ	Fecal SCFA–ADHD SS: ≠; Fecal SCFA–sleep disturbances: ↑; GI features: NA; Diet: NA	NA	Altered fecal SCFA profiles associated with greater ADHD symptom severity and sleep disturbances
Krajewski et al. (2025)	(118)	COH-P	8.9–10.0	87	NR	Urine collection; LC–MS/MS for microbiome-associated metabolites (phenylalanine-, tyrosine-, and L-dopa–derived); urinary heavy metals (As, Cd, Pb, Hg); urinary catecholamines (NA/A ratio); parent- and teacher-reported questionnaires; correlation and path analysis	FBB-ADHS; BRIEF; LSL; SDQ	Microbiome-associated metabolites–ADHD SS: ↑; NA/A ratio–ADHD SS: ↑; Executive dysfunction–metabolites: ↑; GI features: NA; Diet: NA	NA	Higher urinary microbiome-associated metabolites and altered catecholamine ratios associated with worse executive functioning, higher ADHD symptoms, and reduced social behavior
Lawrence et al. (2025)	(119)	RCT	8–13	53 ADHD (NO CG)	DSM-IV/DSM-5	Randomized allocation; fermented dairy (kefir) intervention; parent-reported ADHD rating scales; sleep questionnaires; FSC; gut microbiota sequencing-based analysis	SWAN; Go/NoGo task	Kefir–sleep outcomes: ↑; Kefir–ADHD SS: exploratory; GMC changes post-intervention: ↑; GI features: NA; Diet: intervention only	Kefir supplementation; improvement in sleep-related outcomes; microbiota modulation; ADHD symptom effects exploratory	Fermented dairy intervention modulated gut microbiota and improved sleep outcomes in children with ADHD; effects on core ADHD symptoms were exploratory
Niu et al. (2025)	(120)	INT-OL single arm	NR (children)	42 ADHD	DSM-5	Probiotic supplementation; BRIEF-II; SNAP-IV; repeated-measures ANOVA; adverse event monitoring (CTCAE); blood biochemistry	BRIEF; SNAP-IV	Probiotic–ADHD SS: ↓; Probiotic–executive dysfunction (BRIEF-II): ↓; GI features: NA; Diet: NA	Probiotic mixture (B. animalis BLa80 + L. rhamnosus LRa05, 8 weeks); BRIEF-II GEC ↓; SNAP-IV Inattention ↓; SNAP-IV Hyperactivity–Impulsivity ↓	Probiotic supplementation reduced executive dysfunction and ADHD symptom severity over 8 weeks

**↑**, increase/higher; **↓**, decrease/lower; **≠**, different/altered; **Ø**, no association; **NSA**, non-significant (statistically); **NA**, not assessed; **NR**, not reported; **ASD**, autism spectrum disorder; **ADHD**, attention-deficit/hyperactivity disorder; **CG**, control group; **GI**, gastrointestinal; **GSS**, gastrointestinal symptoms; **ADOS-2**, Autism Diagnostic Observation Schedule, Second Edition; **CARS**, Childhood Autism Rating Scale; **SRS**, Social Responsiveness Scale; **SSP**, Short Sensory Profile; **TRF**, Teacher Report Form; **GSRS**, Gastrointestinal Symptom Rating Scale; **CSHQ**, Children's Sleep Habits Questionnaire; **CGAS**, Children's Global Assessment Scale; **BRIEF**, Behavior Rating Inventory of Executive Function; **BRIEF-II**, Behavior Rating Inventory of Executive Function, Second Edition; **CGI-S** Clinical Global Impressions–Severity; **CGI-I**, Clinical Global Impressions–Improvement; **SNAP-IV**, Swanson, Nolan and Pelham Rating Scale–IV; **SDQ**, Strengths and Difficulties Questionnaire; **LSL**, teacher Checklist of Social and Learning Behaviour; **PedsQL 4.0**, Pediatric Quality of Life Inventory, Version 4.0; **CBCL 6**–18, Child Behavior Checklist, Ages 6–18; **ABCL-AP**, Adult Behavior Checklist–Attention Problems subscale; **CASI-5**, Child and Adolescent Symptom Inventory–5; **ADHD-RS-IV**, Attention-Deficit/Hyperactivity Disorder Rating Scale IV; **CPRS-R**, Conners’ Parent Rating Scale–Revised; Conners-3 Conners, Third Edition; **DIVA 2.0**, Diagnostic Interview for ADHD in Adults, Version 2.0; **K-SADS-PL/KSADS-PL**, Kiddie Schedule for Affective Disorders and Schizophrenia; **ADHS-SB**, German ADHD Self-Rating Scale; **FBB-ADHS**, Foreign Rating Scale for ADHD; **FBB-HKS**, German teacher-rated ADHD symptom checklist; **ARS**, ADHD Rating Scale–IV; **K-ARS**, Korean ADHD Rating Scale; **SWAN**, Strengths and Weaknesses of ADHD Symptoms and Normal Behavior Scale; **WCST**, Wisconsin Card Sorting Test; **WURS-k**, Wender Utah Rating Scale, German short version; **CPT**, Continuous Performance Test; **CDI**, Children's Depression Inventory; **FFQ**, Food Frequency Questionnaire; **BMI**, body mass index; **DSM**, Diagnostic and Statistical Manual of Mental Disorders; **ICD**, International Classification of Diseases; **ICD-10**, International Classification of Diseases, 10th revision; **16S rRNA**, 16S ribosomal RNA gene sequencing; **qPCR**, quantitative polymerase chain reaction; **GC-MS**, gas chromatography–mass spectrometry; **QIIME/QIIME2**, microbiome bioinformatics pipeline; **LEfSe**, Linear discriminant analysis Effect Size; **UniFrac**, unique fraction metric (phylogenetic distance); **Chao1**, species richness estimator; **PCA**, principal component analysis; **PD**, phylogenetic diversity; **BTA**, bioinformatic taxonomic analysis; **GMC**, gut microbiota composition; **OTU**, operational taxonomic unit; **ASV**, amplicon sequence variant; **COH**, cohort study; **COH-P**, prospective; **CS**, cross-sectional study; **CC**, case–control study; **C-CS**, comparative cross-sectional study; **RCT**, randomized controlled trial; **INT-OL**, open-label interventional study.

### Study design distribution

Of the 109 included studies, 89 (81.65%) employed observational designs, including cohort, case-control or cross-sectional design, whereas 20 (18.35%) were interventional reports. Because several interventional publications represented secondary analyses of previously reported randomized trial datasets rather than independent trials, intervention-related study designs were further classified to distinguish unique randomized controlled trials (RCTs) from secondary analytical reports and other non-randomized interventional studies. Accordingly, the interventional literature comprised 3 unique RCTs, 6 secondary analyses derived from randomized trial datasets, and 11 open-label interventional study. This distribution confirms that the evidence base was predominantly observational, while the interventional literature remained limited and methodologically heterogeneous, particularly about the distinction between unique randomized trials, secondary analytical reports, and exploratory open-label studies. Two studies included both ASD and ADHD populations and were therefore relevant to both diagnostic groups, while being counted once in the total number of included studies.

Among the 90 ASD studies, 78 were observational and 12 were interventional. Cross-sectional studies (*n* = 68) were the most frequent, followed by case-control studies (*n* = 9). Interventional evidence in ASD included both randomized and non-randomized designs, specifically 2 randomized secondary reports derived from previously reported trial dataset and 10 open-label studies, 4 of which were explicitly described as pilot investigations; 1 of the open-label reports represented a secondary analysis of a previously reported dataset. Specifically, most cross-sectional and case–control studies compared children with ASD with neurotypical or sibling controls, whereas retrospective studies relied on previously collected biological samples, clinical charts, or registry-based datasets.

Among the 21 ADHD studies, the majority were observational studies (*n* = 13), whereas 8 were interventional, comprising 3 unique RCTs, 4 secondary analyses derived from previously reported randomised trial datasets, and 1 open-label study. Although the ADHD evidence base remained numerically limited overall, it included a proportionally higher share of interventional reports than the ASD literature; however, several of these were secondary analyses of previously reported randomized trial datasets rather than independent trials.

### Methodological approaches across studies

Across both ASD and ADHD studies, case definitions were heterogeneous, and diagnostic ascertainment procedures was not uniformly described across cohorts. Gut microbiota composition was most commonly assessed using 16S rRNA gene sequencing, with a smaller number of studies employing shotgun metagenomic sequencing to achieve higher taxonomic or functional resolution (5, 13).

While 16S-based approaches enabled broad characterization of microbial community structure, the limited taxonomic resolution and variability in targeted hypervariable regions likely contributed to inconsistent findings across studies (10). From a clinical perspective, this methodological heterogeneity limits the immediate translatability of specific microbial signatures into diagnostic or prognostic tools.

Sample collection procedures, DNA extraction protocols, sequencing platforms, and bioinformatic pipelines varied substantially across studies, often without harmonization or cross-validation (11).

Gastrointestinal symptoms were assessed using parent-reported questionnaires, structured clinical interviews, or validated gastrointestinal symptom scales, although the specific instruments used varied substantially across studies (4, 91). ASD symptom severity was evaluated using standardized clinical instruments, most frequently the Childhood Autism Rating Scale (CARS) (121), Autism Behavior Checklist (ABC) (122), and the Social Responsiveness Scale (SRS) (123), thereby enabling correlation analyses between behavioral severity and gastrointestinal or microbiota-related variables (4, 5). In ADHD studies, symptom-related outcomes were more variably operationalized and often relied on parent- or caregiver-reported instruments, with fewer studies incorporating structured multidimensional clinical characterization comparable to that reported in the ASD literature. However, not all observational studies included standardized behavioral measures, thereby limiting the ability to draw clinically meaningful inferences across the full body of evidence.

Dietary intake and feeding behaviors were assessed in a subset of studies through food frequency questionnaires, dietary recalls, or caregiver-reported measures of food selectivity (12, 13). The inconsistent inclusion and operationalization of dietary variables represent a critical methodological limitation, given the strong influence of diet on gut microbiota composition. Clinically, this highlights the risk of misattributing diet-driven microbial differences to neurodevelopmental pathology in the absence of systematic dietary assessment (12).

### Methodological appraisal and risk of bias considerations

Formal methodological appraisal was focused on interventional studies and is summarized in the Supplementary Tables. The 9 reports derived from randomized designs (3 unique RCTs and 6 secondary analyses of previously reported randomized trial datasets) were appraised using the RoB 2 framework. Overall, 3 of the 9 reports were judged at high risk of bias, whereas the remaining 6 raised some concerns, with no randomized report meeting an overall low-risk profile. Across these studies, the most frequent concerns did not primarily arise from randomization procedures or deviations from intended interventions, which were often adequately described, but rather from missing outcome data, selective reporting, and the exploratory nature of secondary or *post hoc* analyses. In particular, secondary analyses derived from parent RCTs frequently relied on non-prespecified outcomes, subgroup analyses, or mechanistic sub studies, limiting their interpretive weight as independent confirmatory evidence.

The 11 non-randomized open-label interventional studies were appraised using MINORS, with scores ranging from 8 to 13 out of 16 (median: 11/16), indicating overall moderate methodological quality consistent with early-phase exploratory designs. The most recurrent strengths were clearly stated aims, prospective data collection in most studies, and endpoints generally appropriate to the exploratory objectives. However, common limitations included the absence of blinded outcome assessment, frequent reliance on parent- or caregiver-reported measures, small sample sizes, incomplete or >5% loss to follow-up in several studies, and the absence of prospective sample size calculation in most reports. Collectively, these findings indicate that the interventional evidence base is methodologically informative but remains constrained by exploratory designs, secondary outcome analyses, and limited protection against performance, attrition, and reporting bias. Accordingly, findings from interventional studies were interpreted cautiously and considered primarily hypothesis-generating, particularly with regard to behavioral outcomes.

Given the predominance of observational designs in the overall evidence base (89/109 studies), no formal study-by-study risk-of-bias tool was applied to the remaining literature because of the marked heterogeneity in design, biological matrices, sequencing platforms, phenotypic characterization, and outcome definitions. Instead, these studies were interpreted through a structured narrative methodological appraisal. Across the observational literature, the most consistent limitations were the predominance of cross-sectional and case–control designs, which precluded temporal or causal inference; frequent use of relatively small or uneven samples, despite some larger cohort-based datasets; and substantial heterogeneity in case definition and diagnostic ascertainment, which was not uniformly described across cohorts. Methodologically, most microbiome studies relied on 16S rRNA sequencing, while a smaller subset used shotgun metagenomics, metabolomics, or non-fecal biological matrices (e.g., urine, plasma, saliva, oral samples, or biopsy-based approaches), resulting in limited comparability across studies. In addition, sample collection procedures, DNA extraction methods, sequencing targets, and bioinformatic pipelines varied considerably, often without harmonization or cross-validation.

From a clinical interpretive perspective, gastrointestinal symptoms and dietary variables were not handled consistently across observational studies. Gastrointestinal features were assessed using heterogeneous approaches, ranging from validated symptom scales to parent-reported questionnaires or were not assessed at all, particularly in ADHD studies. Similarly, dietary intake or food selectivity was systematically examined only in a subset of studies, whereas in many reports diet was either not assessed, not reported, or only indirectly described, despite its likely influence on microbiota-related findings. Finally, not all observational studies incorporated standardized neurobehavioral instruments, and several reported biological group differences without parallel symptom-level correlations, further limiting the strength and clinical translatability of repeated associations. Overall, the observational evidence was therefore interpreted as hypothesis-generating and correlational rather than confirmatory, and any apparent convergence across studies was weighed against the recurrent influence of design-related and measurement-related sources of uncertainty.

#### Associations between ASD symptoms, gastrointestinal features, and diet

Among ASD observational studies employing standardized clinical measures, several reported associations between ASD symptom severity and gastrointestinal symptom burden. Higher scores on the CARS (121) were associated with increased prevalence and severity of constipation, abdominal pain, and diarrhea in preschool children with ASD (4). Similarly, studies using the ABC (122) reported higher behavioral severity scores in children with ASD presenting with clinically significant gastrointestinal symptoms compared with those without gastrointestinal involvement (5). These findings support the clinical relevance of routinely assessing gastrointestinal symptoms in ASD care pathways, as gastrointestinal comorbidity may identify subgroups with greater overall clinical burden and potentially distinct treatment needs (4, 5).

Biological correlates of gastrointestinal dysfunction were also associated to ASD symptom severity. Increased serum zonulin levels, as a marker of intestinal permeability, were positively correlated with autism severity scores measured using the CARS (38). Additional studies reported associations between inflammatory or immune-related markers and greater ASD severity, particularly in children with co-occurring gastrointestinal symptoms (55). From a clinical perspective, these findings suggest that ASD subgroups characterized by gastrointestinal symptoms and biomarkers of altered barrier function or inflammation may warrant more integrated gastroenterological and immunological assessment, although biomarker-driven stratification requires validation in prospective studies (38, 55).

Dietary selectivity and restrictive eating patterns were frequently examined in observational studies. Children with ASD exhibiting higher degrees of food selectivity showed altered gut microbiota composition and reduced microbial diversity, alongside higher ASD symptom severity scores (13). A large multi-omics study demonstrated that dietary patterns significantly mediated associations between ASD diagnosis, gut microbiota composition, and behavioral traits, with adjustment for diet attenuating several microbiota–behavior associations (12). These findings have clinical implications: dietary assessment should be considered a key component of MGBA-informed clinical evaluation in ASD, both to reduce nutritional risk and to support appropriate interpretation of microbiome findings given that diet may confound or modulate microbiota–behavior relationships (12, 13).

Collectively, these studies indicate recurrent associations between gastrointestinal symptoms, dietary patterns, and ASD related symptom severity when measured using standardized clinical measures are available; however, the predominantly cross-sectional nature of most investigations limits causal inference. Accordingly, any clinical translation should prioritize phenotype-informed screening (gastrointestinal symptom burden and dietary selectivity) and prospective evaluation of targeted interventions rather than universal microbiota-directed treatments (4, 12).

#### Associations between ADHD symptoms, gastrointestinal features, and diet

Evidence linking ADHD-related symptoms dimensions to gastrointestinal features and dietary patterns remained limited and methodologically heterogeneous. Cross-sectional analyses reported differences in gut microbial profiles between children with ADHD and typically developing controls, with some taxa associated with inattention and hyperactivity scores (14). However gastrointestinal symptoms were not systematically assessed across studies, limiting interpretation of whether these microbiota findings related to behavioral dimensions of ADHD or to broader gut-related clinical variability.

A population-based cohort study further examined early-life exposures relevant to the gut–brain axis, reporting that maternal and early childhood antibiotic exposure was associated with an increased risk of ADHD diagnosis in offspring (124). Because gastrointestinal symptoms and direct microbiota measures were not assessed, this study should be interpreted as indirect contextual evidence suggesting that microbiota-related perturbations during critical developmental windows may be relevant to ADHD risk, rather than as direct evidence of MGBA-related symptom mechanisms

Dietary factors were explicitly addressed in a limited number of ADHD studies. Exploratory analyses suggest that dietary patterns and nutrient intake may modulate gut microbiota composition and may be associated with ADHD-related behaviors; however, standardized dietary assessments were inconsistently applied, and findings remained largely descriptive (15). The absence of systematic dietary control limits the interpretation of microbiota–behavior associations in ADHD and underscores the need for integrated dietary assessment in future studies.

From a clinical perspective, the available evidence raises the possibility that, similar to ASD, ADHD may encompass subgroups in which gastrointestinal features or diet-related factors interact with behavioral symptom expression. However, the limited number of studies, heterogeneity of methodologies, and the fact that several interventional reports were secondary analyses of previously reported randomized trial datasets rather than independent trials preclude definitive conclusions regarding clinical screening or microbiota-targeted treatment strategies in ADHD at present (14, 124).

### Interventional studies and clinical outcomes

Interventional studies included in this review were conducted in both ASD and ADHD populations and primarily targeted the gut microbiota through probiotic supplementation, dietary modification, or fecal microbiota transplantation (FMT).

In the ASD-focused literature, interventional evidence comprised 12 reports, including2 randomized secondary analysis of a previously reported randomized trial dataset) and 10 open-label studies, 4 of which were explicitly described as pilot investigations; 1 of the open-label reports represented a secondary analysis of a previously reported dataset. Across these ASD interventional studies,     , gastrointestinal outcomes were the most consistently improved domain, with reductions in constipation, abdominal pain, and overall gastrointestinal symptom burden (28, 91).

Several studies employing probiotic or gut-directed interventions reported improvements in gastrointestinal symptom scales (51, 73, 85), whereas effects on core ASD symptoms were less consistent and were generally reported as secondary outcomes (28, 85). In a prospective study of chronically constipated children with ASD, gut mobilization was associated with both improved bowel function and reductions in ASD symptom severity scores, alongside modulation of urinary p-cresol levels, suggesting a link between gastrointestinal improvement and behavioral outcomes (91). Similarly, pilot probiotic studies reported modest changes in behavioral measures, although gastrointestinal outcomes remained the primary and most robust effects (28).

More invasive microbiota-targeted approaches, such as fecal microbiota transplantation, were investigated mainly through trial protocols and early-phase studies (11, 51, 53). Protocol-based RCTs proposed comprehensive assessment of microbiota remodeling alongside behavioral and gastrointestinal outcomes measured using standardized scales including the ABC (122) and CARS (121), although definitive efficacy data remain limited at present (125).

Across interventional studies, behavioral improvements were generally reported as secondary outcomes, were modest in magnitude, and showed substantial inter-individual variability (85, 87). Importantly, behavioral benefits were more frequently observed in subgroups of children presenting with prominent gastrointestinal symptoms at baseline, suggesting a phenotype-specific response to microbiota-targeted interventions (28, 91).

In contrast, the ADHD literature on microbiota–gut–brain axis interactions remain comparatively smaller but proportionally more interventional in orientation. Of the 21 ADHD studies included (Supplementary Table 2), 13 were observational designs (61.90%), whereas 8 were interventional reports (38.10%), including 3 original-data RCTs, 4 secondary analyses of previously reported randomized datasets and 1 open-label interventional study (4.76%). By comparison, within the ASD field only 2 out of 90 studies were randomized secondary reports derived from previously reported randomized trial datasets (2.22%). Thus, although the ADHD evidence base remained numerically limited overall, it included a proportionally higher share of interventional reports than the ASD literature; however, several of these represented secondary analyses rather than independent confirmatory trials.

Observational ADHD studies predominantly employed comparative cross-sectional or case–control methodologies. Several investigations reported altered gut microbial community structure, including reduced alpha diversity in some cohorts and significant beta-diversity shifts across samples (11, 107, 108, 114). Specific taxa differences were described—such as increased Proteobacteria, Enterobacter, Escherichia, Blautia, and Ruminococcus_gnavus_group, alongside reductions in Bifidobacterium, Faecali bacterium, and Akkermansia—yet no reproducible ADHD-specific microbial signature emerged across studies (107, 108, 114). Metabolic profiling further suggested alterations in short-chain fatty acids (SCFAs) and microbiome-associated tryptophan metabolites (12, 104, 110, 114). Reduced plasma or fecal SCFA concentrations were observed in several cohorts; however, associations with core ADHD symptom severity were inconsistent (12, 110). Instead, SCFA alterations were more robustly linked to sleep disturbances, antibiotic exposure history, and dietary patterns than to inattentive or hyperactive–impulsive symptom scores *per se* (12, 110). Markers of intestinal permeability and somatic comorbidity were explored in a subset of studies (12, 110). Elevated serum zonulin levels were associated with greater hyperactivity and social dysfunction, while co-twin control analyses demonstrated increased gastrointestinal and broader physical health problems in children with ADHD compared with non-ADHD co-twins, even after controlling for shared genetic and environmental factors (102, 106). These findings support the notion that, in ADHD, MGBA-related alterations may intersect with somatic vulnerability dimensions rather than exclusively with core attentional symptoms. Interventional studies in ADHD were largely centered on nutraceutical or probiotic approaches (102). Early-life probiotic supplementation was associated with a reduced risk of later ADHD diagnosis in long-term follow-up (101). Broad-spectrum multinutrient supplementation trials demonstrated significant improvements in clinician-rated global functioning and symptom severity, independent of baseline diet quality (112). Combined probiotic–pharmacological approaches showed greater symptom reduction compared with medication alone, whereas single-strain probiotic trials reported good tolerability but limited superiority over placebo (105). Fermented dairy (kefir) interventions were primarily associated with improvements in sleep-related outcomes, with exploratory effects on core ADHD symptoms (116).

Consequently, despite the proportionally greater representation of interventional reports in ADHD compared with ASD, evidence regarding the therapeutic potential of microbiota- or diet-based interventions in ADHD remains insufficient to support clinical recommendations.

### Summary of findings

This structured narrative review synthesizes clinically relevant human evidence on the MGBA in children and adolescents with ASD and ADHD, and should be interpreted as a focused narrative synthesis rather than as a formal systematic review or comprehensive scoping evidence map. The interpretation of the present findings is grounded in the full set of included primary pediatric human studies. Narrative reviews, systematic reviews, and meta-analyses were not considered part of the formal evidence base, but selected background literature was cited where useful to contextualize the broader field (4, 5, 10–14, 91, 124, 125). Overall, the included evidence is characterized by a strong predominance of observational studies, substantial methodological heterogeneity, and a limited number of interventional studies.

Across ASD studies, observational evidence consistently indicates that gastrointestinal symptoms, dietary selectivity, and microbiota-related alterations are associated with greater ASD symptom severity when assessed using standardized clinical scales, such as CARS, ABC, and SRS.

Methodologically, most studies relied on 16S rRNA gene sequencing and varied widely in sample processing, sequencing platforms, and analytical pipelines. Assessment of diet and gastrointestinal symptoms was inconsistent across studies.

Interventional studies in ASD reported consistent improvements in gastrointestinal outcomes following microbiota- or diet-targeted interventions, whereas effects on core ASD symptoms were modest, heterogeneous, and typically secondary outcomes. Behavioral improvements were more frequently observed in children with prominent gastrointestinal symptoms at baseline, supporting a phenotype-specific rather than universal therapeutic effect.

In ADHD, the evidence base remained substantially smaller and more heterogeneous, although it included a proportionally higher share of interventional reports than the ASD literature. However, several ADHD interventional reports represented secondary analyses rather than independent confirmatory trials. Available findings suggest possible links between microbiota-related factors, diet, and broader associated symptom dimensions, including sleep and somatic vulnerability, but current evidence remains insufficient to support clinically actionable microbiota-targeted strategies.

Taken together, the current evidence supports a model in which microbiota–gut–brain interactions act as modulatory factors influencing symptom expression in ASD and ADHD rather than primary causal mechanisms.

## Discussion

This structured narrative review summarizes pediatric human evidence on the MGBA in ASD and ADHD, highlighting both areas of convergence and substantial gaps in current knowledge. Overall, the findings support the relevance of MGBA-related mechanisms as plausible modulatory factors associated with neurodevelopmental symptom expression; however, it remains unclear whether these mechanisms operate in a predominantly protective, compensatory, or predictive manner with respect to symptom onset and severity. This uncertainty reflects not only the biological complexity of MGBA-related pathways, but also important methodological and conceptual limitations that currently constrain clinical translation. From a clinical perspective, the marked heterogeneity across studies continues to limit the translatability of specific microbial signatures into diagnostic or prognostic tools.

Interpreting main findings, we can resume that in ASD, the accumulated evidence—largely derived from observational studies—consistently indicates associations between gastrointestinal symptom burden, dietary patterns, gut microbiota composition, and greater severity of core behavioral symptoms (4, 12, 67, 74, 90). These associations appear more evident in studies employing standardized clinical scales and are particularly pronounced in subgroups of children with clinically significant gastrointestinal comorbidities (4, 72, 90). This pattern supports the concept of biologically and clinically meaningful ASD sub-phenotypes, in which gut-related factors may interact with neurobehavioral manifestations rather than act as primary causal drivers. Indeed, the MGBA operates through multiple interconnected pathways, including neuroanatomical (vagal and spinal afferent signaling), neuroendocrine (hypothalamic-pituitary-adrenal axis), immunological (cytokine-mediated signaling), and metabolic pathways (microbial-derived metabolites), thereby providing biologically plausible pathways through which gut microbes may be linked to brain development, function, and behaviour (77). In addition, alteration in gut microbial composition have been linked to impaired intestinal barrier function, contributing to more severe gastrointestinal symptoms and a systemic inflammatory response, which in turn has been hypothesized to be associated with altered blood-brain barrier permeability and neurodevelopmental processes.

Interventional studies further suggest that microbiota or diet targeted approaches are more consistently associated with improvements in gastrointestinal symptoms, while effects on core ASD symptoms are generally modest, heterogeneous, and frequently reported as secondary outcomes (28, 51, 73, 85, 87).

Importantly, improvements in broader behavioral or associated clinical dimensions appear more evident in children with prominent gastrointestinal symptoms at baseline, whereas effects on core ASD symptoms remain limited and variable, reinforcing the hypothesis that therapeutic responsiveness may depend on individual clinical profiles rather than a uniform ASD phenotype (37, 51, 53, 91). These broader changes may reflect indirect benefits on associated symptom burden or overall clinical regulation rather than direct modification of core ASD symptom domains.

In contrast, the specific ADHD literature about the association of gastrointestinal symptoms, diet, and behaviour phenotype, remains still limited both in quantity as in methodological depth as well. Although the overall evidence base is still small and heterogeneous, and many findings remain exploratory, and characterized by small sample sizes and heterogeneous outcome measures. While preliminary findings suggest potential links between microbiota-related factors, early-life exposures, diet, and ADHD-related behaviors (63, 77, 104, 106), the current evidence base is insufficient to support definitive mechanistic conclusions or clinical recommendations.

Current findings suggest that MGBA research in ASD has been primarily oriented toward the identification of candidate biomarkers and mechanistic pathways potentially associated with symptom expression. Notably, a higher proportion of nutraceutical or probiotic supplementation approaches were identified in ADHD studies (104, 106, 108, 114, 115, 119), compared to ASD studies, highlighting a relative imbalance in research efforts addressing the role of MGBA in ASD and in ADHD. Within the ASD field, investigations of the gut–brain axis are primarily oriented toward the identification of potential pathogenetic biomarkers and mechanistic pathways to manage ASD core- symptoms (29, 36, 39, 65, 81, 95, 98).

In contrast, in ADHD research, the use of nutraceutical interventions appears to be predominantly associated with changes in associated symptom dimensions and broader functional outcomes, including sleep disturbances, anxiety, and related measures, rather than with consistent improvement in core ADHD symptoms such as inattention, hyperactivity, or impulsivity (15, 119, 120). When reported, gastrointestinal outcomes should likewise be interpreted separately from disorder-specific symptom change, as improvement in gastrointestinal burden or associated symptoms does not necessarily imply modification of the core neurodevelopmental phenotype.

Across the interventional literature, outcomes are more appropriately interpreted across distinct clinical domains rather than as equivalent indicators of treatment response. First, gastrointestinal outcomes appear to be the most consistently improved domain across microbiota- or diet-targeted interventions, particularly in ASD, where reductions in gastrointestinal symptom burden were more reproducibly reported than changes in neurobehavioral endpoints (28, 41, 51, 73, 85, 91). Second, effects on core neurodevelopmental symptoms remain limited and heterogeneous in both disorders. In ASD, changes in core symptom domains were generally modest, variable, and often reported as secondary outcomes (28, 51, 73, 85, 87), while in ADHD the available evidence remains too limited and methodologically heterogeneous to support consistent effects on core symptoms such as inattention, hyperactivity, or impulsivity (15, 104, 106, 108, 114, 115, 119). Third, some studies reported improvements in associated symptom dimensions, including sleep disturbances, anxiety-related symptoms, irritability, or broader behavioral dysregulation, particularly in clinically selected subgroups or in children with prominent gastrointestinal symptoms at baseline (15, 37, 51, 53, 91, 119, 120). These changes may reflect indirect benefits on associated symptom burden or overall clinical regulation rather than direct modification of core neurodevelopmental symptom domains. Finally, broader functional outcomes, when reported, should be interpreted separately from both core and associated symptom changes, as global functioning or parent-reported clinical improvement may capture non-specific or downstream effects that do not necessarily indicate disorder-specific therapeutic efficacy. This distinction is particularly relevant in child and adolescent psychiatry, where symptom expression and treatment response are strongly shaped by developmental level, comorbidity burden, and environmental context.

Some methodological considerations and research gaps should be acknowledged. First, by restricting the present synthesis to pediatric human studies, we prioritized clinical relevance and translational applicability, but this necessarily reduced the breadth of mechanistic evidence that could be considered, particularly with regard to preclinical MGBA pathways. Therefore, biological interpretations should remain cautious and should not be regarded as mechanistic confirmation. In addition, the available literature was markedly heterogeneous in terms of study design, phenotypic characterization, sequencing methods, dietary exposures, gastrointestinal symptom burden, intervention protocols, and outcome measures. Dietary factors deserve particular methodological caution, as they were frequently invoked as relevant confounders but were not consistently or rigorously handled across the included studies. In many reports, dietary intake, food selectivity, or feeding-related behaviors were assessed using non-standardized parent-reported measures, broad clinical descriptors, or incompletely described dietary exclusion criteria rather than structured nutritional assessment. In addition, few studies systematically controlled for dietary composition, restrictive eating patterns, supplementation history, or concurrent dietary interventions when interpreting microbiota-related findings. This limitation is particularly relevant in ASD, where food selectivity and feeding difficulties are common and may independently affect both microbiota profiles and behavioral presentation, thereby complicating interpretation of MGBA-related associations (77, 111). This heterogeneity also affected the interpretability of intervention outcomes, which required cautious domain-specific consideration within the narrative synthesis, and limited comparability across studies, thereby constraining the possibility of more formal stratified analyses within the scope of the present review. For this reason, findings were synthesized using a structured narrative approach, while interventional studies were further interpreted through formal methodological appraisal, including RoB 2 for reports derived from randomized designs, MINORS for open-label interventional studies, and narrative methodological consideration of the predominantly observational literature. Overall, the methodological approaches used across studies, support the exploratory characterization of microbiota–gut–brain interactions but currently lack the standardization required for clinical implementation.

Across both ASD and ADHD, many studies have used cross-sectional and/or retrospective designs, which preclude causal inference and limit understanding of temporal relationships between microbiota alterations and neurodevelopmental outcomes. Accordingly, observed associations between gut-related factors and neurobehavioral features should be interpreted as correlational rather than causal, and should not be taken as evidence that MGBA-related alterations directly determine symptom onset, severity, or treatment response. Sample sizes are frequently modest, and methodological inter-study heterogeneity—particularly in microbiome sequencing approaches, dietary assessment, and gastrointestinal phenotyping—complicates comparison across studies and contributes to inconsistent findings, underscoring the need for standardized methodologies before microbiota-based clinical applications can be reliably considered.

From a child and adolescent psychiatry perspective, additional clinically relevant sources of heterogeneity should also be considered when interpreting MGBA-related findings (77, 111). Developmental and cognitive level may influence dietary selectivity, treatment adherence, caregiver-reported symptom burden, and performance on standardized behavioral scales, thereby affecting the apparent relationship between gut-related factors and neurobehavioral outcomes (126, 127). Likewise, common associated dimensions such as sensory abnormalities, feeding difficulties, sleep disturbances, and anxiety or mood symptoms may independently affect both gastrointestinal function and microbiota-related profiles, while also shaping behavioral presentation (77, 111, 119). In ADHD particularly, medication exposure represents a further important confounder, as stimulant and non-stimulant treatments may alter appetite, weight trajectory, sleep, and gastrointestinal symptoms, thereby complicating interpretation of MGBA-related associations (107, 110). These factors reinforce the need to interpret microbiota-related findings within a broader developmental psychopathology framework, in which biological, behavioral, and environmental factors interact to shape symptom expression.

Future research should prioritize well-designed prospective longitudinal studies with larger and more representative sample sizes to clarify the directionality and stability of MGBA-related associations. In addition, RCTs employing standardized microbiome methodologies, rigorous dietary control, and clearly defined clinical endpoints are essential to determine whether microbiota- or diet-targeted interventions can meaningfully influence neurodevelopmental symptoms beyond gastrointestinal outcomes.

Despite these limitations, the current evidence carries cautious clinical implications. In ASD, assessment of gastrointestinal symptoms and dietary patterns should be routinely considered, as these factors are frequently associated with greater symptom severity and may identify subgroups more likely to benefit from more individualized assessment and targeted adjunctive interventions (12, 37, 41, 63, 77).

Interventions targeting the gut ecosystem through dietary modification, probiotics, prebiotics, or microbiota transplants may be associated with reduction in gastrointestinal symptom burden and in selected cases, may indirectly support improvements in associated behavioral or functional dimensions rather than consistent change in core ASD symptoms (28, 41, 51, 73, 85, 91).

However, the current evidence does not support these approaches as stand-alone therapeutic strategies for core neurodevelopmental symptoms, and any clinical use should be considered exploratory, adjunctive and individualized rather than universally applied.

For ADHD, potential clinical implications remain largely hypothetical, and the available evidence is insufficient to support microbiota-targeted interventions as clinically established therapeutic options. While MGBA-related mechanisms may eventually inform novel therapeutic strategies, the current evidence base remains preliminary and heterogeneous, limiting confident clinical implementation. Although outside the predefined scope of this pediatric-focused review, selected adult and meta-analytic literature, including an adult double-blind RCT in ADHD and a recent pooled analysis of microbiota-targeted interventions (128, 129), may provide broader contextual support for the hypothesis that such approaches could exert modest, symptom-specific effects. However, these studies were not part of the formal evidence base of the present review and should be interpreted only as contextual background rather than as direct support for the pediatric conclusions presented here. More rigorous research is required before translating microbiota- or diet-based interventions into ADHD clinical practice.

## Conclusions

In conclusion, this structured narrative review highlights a growing body of pediatric human evidence suggesting that the microbiota–gut–brain axis may be relevant to symptom expression and gastrointestinal-related clinical features in ASD, with emerging but still limited and heterogeneous evidence in ADHD. While MGBA-related interventions show promise—particularly for alleviating gastrointestinal symptoms and potentially modulating behavioral outcomes in selected ASD subgroups—definitive conclusions regarding therapeutic efficacy remain premature. Advancing the field will require coordinated efforts to conduct large-scale prospective studies and RCTs with harmonized methodologies and clinically meaningful outcomes. Such approaches are essential to determine whether MGBA-targeted strategies can ultimately play a role in reducing neurodevelopmental symptom burden in ASD and, potentially, in ADHD.

## References

[1] Lord C Brugha TS Charman T Cusack J Dumas G Frazier T Autism spectrum disorder. Nat Rev Dis Primers. (2020) 6(1):5. 10.1038/s41572-019-0138-4 31949163 PMC8900942

[2] Thapar A Cooper M Rutter M. Neurodevelopmental disorders. Lancet Psychiatry. (2017) 4(4):339–46. 10.1016/S2215-0366(16)30376-5 PubMed PMID: 27979720. 27979720

[3] Cai RY Samson AC Uljarevic M. Editorial: emotion regulation in neurodevelopmental disorders: current understanding and treatments. Front Psychiatry. (2024) 15:1467834. 10.3389/fpsyt.2024.1467834 PubMed PMID: 39143960; PubMed Central PMCID: PMC11322352. 39143960 PMC11322352

[4] Fulceri F Morelli M Santocchi E Cena H Del Bianco T Narzisi A Gastrointestinal symptoms and behavioral problems in preschoolers with autism Spectrum disorder. Dig Liver Dis. (2016) 48(3):248–54. 10.1016/j.dld.2015.11.026 26748423

[5] Rose DR Yang H Serena G Sturgeon C Ma B Careaga M Differential immune responses and microbiota profiles in children with autism spectrum disorders and co-morbid gastrointestinal symptoms. Brain Behav Immun. (2018) 70:354–68. 10.1016/j.bbi.2018.03.025 29571898 PMC5953830

[6] Hamamah S Aghazarian A Nazaryan A Hajnal A Covasa M. Role of Microbiota-gut-brain axis in regulating dopaminergic signaling. Biomedicines. (2022) 10(2):436. 10.3390/biomedicines10020436 PubMed PMID: 35203645; PubMed Central PMCID: PMC8962300. 35203645 PMC8962300

[7] Charitos IA Inchingolo AM Ferrante L Inchingolo F Inchingolo AD Castellaneta F The gut microbiota’s role in neurological, psychiatric, and neurodevelopmental disorders. Nutrients. (2024) 16(24):4404. 10.3390/nu16244404 PubMed PMID: 39771025; PubMed Central PMCID: PMC11677138. 39771025 PMC11677138

[8] Cryan JF O’Riordan KJ Cowan CSM Sandhu KV Bastiaanssen TFS Boehme M The Microbiota-gut-brain axis. Physiol Rev. (2019) 99(4):1877–2013. 10.1152/physrev.00018.2018 31460832

[9] Sharon G Sampson TR Geschwind DH Mazmanian SK. The central nervous system and the gut microbiome. Cell. (2016) 167(4):915–32. 10.1016/j.cell.2016.10.027 27814521 PMC5127403

[10] Iglesias-Vázquez L Van Ginkel Riba G Arija V Canals J. Composition of gut Microbiota in children with autism Spectrum disorder: a systematic review and meta-analysis. Nutrients. (2020) 12(3):792. 10.3390/nu12030792 32192218 PMC7146354

[11] Nirmalkar K Qureshi F Kang DW Hahn J Adams JB Krajmalnik-Brown R. Shotgun metagenomics study suggests alteration in Sulfur metabolism and oxidative stress in children with autism and improvement after Microbiota transfer therapy. Int J Mol Sci. (2022) 23(21):13481. 10.3390/ijms232113481 36362265 PMC9654974

[12] Yap CX Henders AK Alvares GA Wood DLA Krause L Tyson GW Autism-related dietary preferences mediate autism-gut microbiome associations. Cell. (2021) 184(24):5916–5931.e17. 10.1016/j.cell.2021.10.015 34767757

[13] Tomova A Soltys K Kemenyova P Karhanek M Babinska K. The influence of food intake specificity in children with autism on gut Microbiota. Int J Mol Sci. (2020) 21(8):2797. 10.3390/ijms21082797 32316625 PMC7215614

[14] Prehn-Kristensen A Zimmermann A Tittmann L Lieb W Schreiber S Baving L Reduced microbiome alpha diversity in young patients with ADHD. PLoS One. (2018) 13(7):e0200728. 10.1371/journal.pone.0200728 30001426 PMC6042771

[15] Boonchooduang N Louthrenoo O Likhitweerawong N Kunasol C Nawara W Thonusin C Gut Microbiota-derived short-chain fatty acids and sleep disturbances in pediatric attention-deficit/hyperactivity disorder: insights into neurobiological links and treatment implications. Pediatr Neurol. (2025) 172:8–14. 10.1016/j.pediatrneurol.2025.07.017 PubMed PMID: 40845726. 40845726

[16] Solek CM Farooqi N Verly M Lim TK Ruthazer ES. Maternal immune activation in neurodevelopmental disorders. Dev Dyn. (2018) 247(4):588–619. 10.1002/dvdy.24612 29226543

[17] Sotgiu S Manca S Gagliano A Minutolo A Melis MC Pisuttu G Immune regulation of neurodevelopment at the mother–foetus interface: the case of autism. Clin Transl Immunology. (2020) 9(11):e1211. 10.1002/cti2.1211 33209302 PMC7662086

[18] Page MJ McKenzie JE Bossuyt PM Boutron I Hoffmann TC Mulrow CD The PRISMA 2020 statement: an updated guideline for reporting systematic reviews. Syst Rev. (2021) 10(1):89. 10.1186/s13643-021-01626-4 33781348 PMC8008539

[19] Açıkel SB Kara A Bağcı Z Can Ü. Serum trimethylamine N-oxide and lipopolysaccharide binding protein levels among children diagnosed with autism spectrum disorder. Int J Dev Neurosci. (2023) 83(6):571–7. 10.1002/jdn.10287 37525434

[20] Ahmed SA Elhefnawy AM Azouz HG Roshdy YS Ashry MH Ibrahim AE Study of the gut microbiome profile in children with autism Spectrum disorder: a single tertiary hospital experience. J Mol Neurosci. (2020) 70(6):887–96. 10.1007/s12031-020-01500-3 32062762

[21] Alookaran J Liu Y Auchtung TA Tahanan A Hessabi M Asgarisabet P Fungi: friend or foe? A mycobiome evaluation in children with autism and gastrointestinal symptoms. J Pediatr Gastroenterol Nutr. (2022) 74(3):377–82. 10.1097/MPG.0000000000003349 34724444 PMC8885784

[22] Alshammari MK AlKhulaifi MM Al Farraj DA Somily AM Albarrag AM. Incidence of Clostridium perfringens and its toxin genes in the gut of children with autism spectrum disorder. Anaerobe. (2020) 61:102114. 10.1016/j.anaerobe.2019.102114 31704282

[23] Anwar A Marini M Abruzzo PM Bolotta A Ghezzo A Visconti P Quantitation of plasma thiamine, related metabolites and plasma protein oxidative damage markers in children with autism spectrum disorder and healthy controls. Free Radic Res. (2016) 50(sup1):S85–90. 10.1080/10715762.2016.1239821 27667096

[24] Averina OV Kovtun AS Polyakova SI Savilova AM Rebrikov DV Danilenko VN. The bacterial neurometabolic signature of the gut microbiota of young children with autism spectrum disorders. J Med Microbiol. (2020) 69(4):558–71. 10.1099/jmm.0.001178 32213246

[25] Belardo A Gevi F Zolla L. The concomitant lower concentrations of vitamins B6, B9 and B12 may cause methylation deficiency in autistic children. J Nutr Biochem. (2019) 70:38–46. 10.1016/j.jnutbio.2019.04.004 31151052

[26] Bent S Lawton B Warren T Widjaja F Dang K Fahey JW Identification of urinary metabolites that correlate with clinical improvements in children with autism treated with sulforaphane from broccoli. Mol Autism. (2018) 9(1):35. 10.1186/s13229-018-0218-4 29854372 PMC5975568

[27] Bhusri B Sutheeworapong S Kittichotirat W Kusonmano K Thammarongtham C Lertampaiporn S Characterization of gut microbiota on gender and age groups bias in Thai patients with autism spectrum disorder. Sci Rep. (2025) 15(1):2587. 10.1038/s41598-025-86740-2 39833480 PMC11747245

[28] Billeci L Callara AL Guiducci L Prosperi M Morales MA Calderoni S A randomized controlled trial into the effects of probiotics on electroencephalography in preschoolers with autism. Autism. (2023) 27(1):117–32. 10.1177/13623613221082710 PubMed PMID: 35362336; PubMed Central PMCID: PMC9806478. 35362336 PMC9806478

[29] Chamtouri M Merghni A Salazar N Redruello B Gaddour N Mastouri M An overview on fecal profiles of amino acids and related amino-derived compounds in children with autism Spectrum disorder in Tunisia. Molecules. (2023) 28(7):3269. 10.3390/molecules28073269 37050030 PMC10096484

[30] Chen Y Fang H Li C Wu G Xu T Yang X Gut Bacteria shared by children and their mothers associate with developmental level and social deficits in autism Spectrum disorder. mSphere. (2020) 5(6):e01044–20. 10.1128/mSphere.01044-20 33268567 PMC7716279

[31] Chen S Xue B Zhou R Qian A Tao J Yang C Abnormal stability of dynamic functional architecture in drug-naïve children with attention-deficit/hyperactivity disorder. BMC Psychiatry. (2024) 24(1):851. 10.1186/s12888-024-06310-0 39592983 PMC11590522

[32] Chen H( Yi B Ma Z(. Resilience of human gut microbiomes in autism spectrum disorder: measured using stiffness network analysis. Microbiol Spectr. (2025) 13(3):e01078–24. 10.1128/spectrum.01078-24 PMC11878074 39902951

[33] Chiappori F Cupaioli FA Consiglio A Di Nanni N Mosca E Licciulli VF Analysis of faecal Microbiota and small ncRNAs in autism: detection of miRNAs and piRNAs with possible implications in host–gut Microbiota cross-talk. Nutrients. (2022) 14(7):1340. 10.3390/nu14071340 35405953 PMC9000903

[34] Cuomo M Coretti L Costabile D Della Monica R De Riso G Buonaiuto M Host fecal DNA specific methylation signatures mark gut dysbiosis and inflammation in children affected by autism spectrum disorder. Sci Rep. (2023) 13(1):18197. 10.1038/s41598-023-45132-0 37875530 PMC10598023

[35] Daneberga Z Nakazawa-Miklasevica M Berga-Svitina E Murmane D Isarova D Cupane L Urinary organic acids spectra in children with altered gut microbiota composition and autistic spectrum disorder. Nord J Psychiatry. (2022) 76(7):523–9. 10.1080/08039488.2021.2014954 34935590

[36] De Sales-Millán A Reyes-Ferreira P Aguirre-Garrido JF Corral-Guillé I Barrientos-Ríos R Velázquez-Aragón JA. Comprehensive analysis of gut Microbiota composition and functional metabolism in children with autism Spectrum disorder and neurotypical children: implications for sex-based differences and metabolic dysregulation. Int J Mol Sci. (2024) 25(12):6701. 10.3390/ijms25126701 38928411 PMC11203636

[37] Di Benedetto G Sorge G Sarchiapone M Di Martino L. Dietary patterns, not gut microbiome composition, are associated with behavioral challenges in children with autism: an observational study. Nutrients. (2025) 17(21):3476. 10.3390/nu17213476 41228547 PMC12610858

[38] Esnafoglu E Cırrık S Ayyıldız SN Erdil A Ertürk EY Daglı A Increased Serum zonulin levels as an intestinal permeability marker in autistic subjects. J Pediatr. (2017) 188:240–4. 10.1016/j.jpeds.2017.04.004 PubMed PMID: 28502607. 28502607

[39] Fiorentino M Sapone A Senger S Camhi SS Kadzielski SM Buie TM Blood–brain barrier and intestinal epithelial barrier alterations in autism spectrum disorders. Mol Autism. (2016) 7(1):49. 10.1186/s13229-016-0110-z 27957319 PMC5129651

[40] Gabriele S Sacco R Altieri L Neri C Urbani A Bravaccio C Slow intestinal transit contributes to elevate urinary p -cresol level in I talian autistic children. Autism Res. (2016) 9(7):752–9. 10.1002/aur.1571 26437875

[41] Gaougaou G Zahra R Morel S Bélanger V Knoth IS Cousineau D Acceptability and safety of a probiotic beverage supplementation (bio-K +) and feasibility of the proposed protocol in children with a diagnosis of autism spectrum disorder. J Neurodev Disord. (2025) 17(1):30. 10.1186/s11689-025-09617-5 40413384 PMC12102953

[42] Gevi F Zolla L Gabriele S Persico AM. Urinary metabolomics of young Italian autistic children supports abnormal tryptophan and purine metabolism. Mol Autism. (2016) 7(1):47. 10.1186/s13229-016-0109-5 27904735 PMC5121959

[43] Gevi F Belardo A Zolla L. A metabolomics approach to investigate urine levels of neurotransmitters and related metabolites in autistic children. Biochim Biophys Acta Mol Basis Dis. (2020) 1866(10):165859. 10.1016/j.bbadis.2020.165859 32512190

[44] Góra B Gofron Z Grosiak M Aptekorz M Kazek B Kocelak P Toxin profile of fecal Clostridium perfringens strains isolated from children with autism spectrum disorders. Anaerobe. (2018) 51:73–7. 10.1016/j.anaerobe.2018.03.005 29526827

[45] Inoue R Sakaue Y Sawai C Sawai T Ozeki M Romero-Pérez GA A preliminary investigation on the relationship between gut microbiota and gene expressions in peripheral mononuclear cells of infants with autism spectrum disorders. Biosci Biotechnol Biochem. (2016) 80(12):2450–8. 10.1080/09168451.2016.1222267 27581276

[46] Jendraszak M Gałęcka M Kotwicka M Regdos A Pazgrat-Patan M Andrusiewicz M. Commercial microbiota test revealed differences in the composition of intestinal microorganisms between children with autism spectrum disorders and neurotypical peers. Sci Rep. (2021) 11(1):24274. 10.1038/s41598-021-03794-8 34931007 PMC8688445

[47] Jory J. Abnormal fatty acids in Canadian children with autism. Nutrition. (2016) 32(4):474–7. 10.1016/j.nut.2015.10.019 26746679

[48] Józefczuk J Konopka E Bierła JB Trojanowska I Sowińska A Czarnecki R The occurrence of antibodies against gluten in children with autism Spectrum disorders does not correlate with serological markers of impaired intestinal permeability. J Med Food. (2018) 21(2):181–7. 10.1089/jmf.2017.0069 29072974

[49] Jung Y Lee T Oh HS Hyun Y Song S Chun J Gut microbial and clinical characteristics of individuals with autism spectrum disorder differ depending on the ecological structure of the gut microbiome. Psychiatry Res. (2024) 335:115775. 10.1016/j.psychres.2024.115775 38503005

[50] Kandeel WA Meguid NA Bjørklund G Eid EM Farid M Mohamed SK Impact of Clostridium Bacteria in children with autism Spectrum disorder and their anthropometric measurements. J Mol Neurosci. (2020) 70(6):897–907. 10.1007/s12031-020-01482-2 32130666

[51] Kang DW Adams JB Gregory AC Borody T Chittick L Fasano A Microbiota transfer therapy alters gut ecosystem and improves gastrointestinal and autism symptoms: an open-label study. Microbiome. (2017) 5:10. 10.1186/s40168-016-0225-7 PubMed PMID: 28122648; PubMed Central PMCID: PMC5264285. 28122648 PMC5264285

[52] Kang DW Ilhan ZE Isern NG Hoyt DW Howsmon DP Shaffer M Differences in fecal microbial metabolites and microbiota of children with autism spectrum disorders. Anaerobe. (2018) 49:121–31. 10.1016/j.anaerobe.2017.12.007 29274915

[53] Kang DW Adams JB Coleman DM Pollard EL Maldonado J McDonough-Means S Long-term benefit of Microbiota transfer therapy on autism symptoms and gut microbiota. Sci Rep. (2019) 9(1):5821. 10.1038/s41598-019-42183-0 30967657 PMC6456593

[54] Kantarcioglu AS Kiraz N Aydin A. Microbiota-Gut-Brain axis: yeast Species isolated from stool samples of children with suspected or diagnosed autism Spectrum disorders and *in vitro* susceptibility against nystatin and fluconazole. Mycopathologia. (2016) 181(1–2):1–7. 10.1007/s11046-015-9949-3 PubMed PMID: 26442855. 26442855

[55] Karagözlü S Dalgıç B İşeri E. The relationship of severity of autism with gastrointestinal symptoms and Serum zonulin levels in autistic children. J Autism Dev Disord. (2022) 52(2):623–9. 10.1007/s10803-021-04966-1 33743117

[56] Kartalcı G Çalışkan Demir A Kartalcı Ş Üremiş N Türköz Y. Evaluation of blood zonulin levels, inflammatory processes and neuronal changes in children with autism spectrum disorder. Psychiatr Danub. (2022) 34(2):279–87. 10.24869/psyd.2022.279 35772138

[57] Khalil M Azouz HG Ahmed SA Gad HA Omar OM. Sensory processing and gastrointestinal manifestations in autism Spectrum disorders: no relation to Clostridium difficile. J Mol Neurosci. (2021) 71(1):153–61. 10.1007/s12031-020-01636-2 32607756

[58] Kim JI Kim BN Lee YA Shin CH Hong YC Lim YH. The mediating role of the gut microbiome in the association between ambient air pollution and autistic traits. Int J Hyg Environ Health. (2022) 246:114047. 10.1016/j.ijheh.2022.114047 36215749

[59] Mahdi ES Komijani M Alaghmand A. Metagenomics study suggests the role of vitamins and gut microbiome in autism Spectrum disorder. Digestion. (2025) 106(6):515–29. 10.1159/000545483 40300566

[60] Kong X Liu J Cetinbas M Sadreyev R Koh M Huang H New and preliminary evidence on altered oral and gut Microbiota in individuals with autism Spectrum disorder (ASD): implications for ASD diagnosis and subtyping based on microbial biomarkers. Nutrients. (2019) 11(9):2128. 10.3390/nu11092128 PubMed PMID: 31489949; PubMed Central PMCID: PMC6770733. 31489949 PMC6770733

[61] Kong X Liu J Liu K Koh M Tian R Hobbie C Altered autonomic functions and gut microbiome in individuals with autism Spectrum disorder (ASD): implications for assisting ASD screening and diagnosis. J Autism Dev Disord. (2021) 51(1):144–57. 10.1007/s10803-020-04524-1 32410097

[62] Kovtun AS Averina OV Alekseeva MG Danilenko VN. Antibiotic resistance genes in the gut Microbiota of children with autistic Spectrum disorder as possible predictors of the disease. Microb Drug Resist. (2020) 26(11):1307–20. 10.1089/mdr.2019.0325 31916894

[63] Kurokawa S Nomura K Sanada K Miyaho K Ishii C Fukuda S A comparative study on dietary diversity and gut microbial diversity in children with autism spectrum disorder, attention-deficit hyperactivity disorder, their neurotypical siblings, and non-related neurotypical volunteers: a cross-sectional study. J Child Psychol Psychiatry. (2024) 65(9):1184–95. 10.1111/jcpp.13962 38562118

[64] Laue HE Korrick SA Baker ER Karagas MR Madan JC. Prospective associations of the infant gut microbiome and microbial function with social behaviors related to autism at age 3 years. Sci Rep. (2020) 10(1):15515. 10.1038/s41598-020-72386-9 32968156 PMC7511970

[65] Li N Yang J Zhang J Liang C Wang Y Chen B Correlation of gut microbiome between ASD children and mothers and potential biomarkers for risk assessment. Genom Proteom Bioinform. (2019) 17(1):26–38. 10.1016/j.gpb.2019.01.002 PMC6520911 31026579

[66] Liu J Liu X Xiong XQ Yang T Cui T Hou NL Effect of vitamin A supplementation on gut microbiota in children with autism spectrum disorders - a pilot study. BMC Microbiol. (2017) 17(1):204. 10.1186/s12866-017-1096-1 28938872 PMC5610466

[67] Liu S Li E Sun Z Fu D Duan G Jiang M Altered gut microbiota and short chain fatty acids in Chinese children with autism spectrum disorder. Sci Rep. (2019) 9(1):287. 10.1038/s41598-018-36430-z 30670726 PMC6342986

[68] Liu Z Mao X Dan Z Pei Y Xu R Guo M Gene variations in autism Spectrum disorder are associated with alternation of gut microbiota, metabolites and cytokines. Gut Microbes. (2021) 13(1):1854967. 10.1080/19490976.2020.1854967 33412999 PMC7808426

[69] Lussu M Noto A Masili A Rinaldi AC Dessì A De Angelis M The urinary 1H-NMR metabolomics profile of an Italian autistic children population and their unaffected siblings. Autism Res. (2017) 10(6):1058–66. 10.1002/aur.1748 28296209

[70] Marler S Ferguson BJ Lee EB Peters B Williams KC McDonnell E Brief report: whole blood serotonin levels and gastrointestinal symptoms in autism Spectrum disorder. J Autism Dev Disord. (2016) 46(3):1124–30. 10.1007/s10803-015-2646-8 26527110 PMC4852703

[71] Marler S Ferguson BJ Lee EB Peters B Williams KC McDonnell E Association of rigid-compulsive behavior with functional constipation in autism Spectrum disorder. J Autism Dev Disord. (2017) 47(6):1673–81. 10.1007/s10803-017-3084-6 28289979 PMC5526215

[72] Martínez-González AE Cervin M Pérez-Sánchez S. Assessing gastrointestinal symptoms in people with autism: applying a new measure based on the Rome IV criteria. Dig Liver Dis. (2024) 56(11):1863–70. 10.1016/j.dld.2024.05.019 38851976

[73] Meguid NA Mawgoud YIA Bjørklund G Mehanne NS Anwar M Effat BAEK Molecular characterization of probiotics and their influence on children with autism Spectrum disorder. Mol Neurobiol. (2022) 59(11):6896–902. 10.1007/s12035-022-02963-8 36050597

[74] Nalbant K Erden S Yazar A Kılınç İ. investigation of the relation between epithelial barrier function and autism symptom severity in children with autism Spectrum disorder. J Mol Neurosci. (2022) 72(4):741–7. 10.1007/s12031-021-01954-z 34988901

[75] Osredkar J Fabjan T Godnov U Jekovec-Vrhovšek M Giebułtowicz J Bobrowska-Korczak B Systemic uremic toxin burden in autism Spectrum disorder: a stratified urinary metabolite analysis. Int J Mol Sci. (2025) 26(15):7070. 10.3390/ijms26157070 40806203 PMC12345674

[76] Palkova L Tomova A Repiska G Babinska K Bokor B Mikula I Evaluation of 16S rRNA primer sets for characterisation of microbiota in paediatric patients with autism spectrum disorder. Sci Rep. (2021) 11(1):6781. 10.1038/s41598-021-86378-w 33762692 PMC7991656

[77] Petropoulos A Anesiadou S Michou M Lymperatou A Roma E Chrousos G Functional gastrointestinal symptoms in children with autism and ADHD: profiles of hair and salivary cortisol, Serum leptin concentrations and externalizing/internalizing problems. Nutrients. (2024) 16(10):1538. 10.3390/nu16101538 PubMed PMID: 38794776; PubMed Central PMCID: PMC11124526. 38794776 PMC11124526

[78] Plaza-Díaz J Gómez-Fernández A Chueca N Torre-Aguilar MJDL Gil Á Perez-Navero JL Autism Spectrum disorder (ASD) with and without mental regression is associated with changes in the fecal Microbiota. Nutrients. (2019) 11(2):337. 10.3390/nu11020337 30764497 PMC6412819

[79] Qiao Y Wu M Feng Y Zhou Z Chen L Chen F. Alterations of oral microbiota distinguish children with autism spectrum disorders from healthy controls. Sci Rep. (2018) 8(1):1597. 10.1038/s41598-018-19982-y 29371629 PMC5785483

[80] Quan L Yi J Zhao Y Zhang F Shi XT Feng Z Plasma trimethylamine N-oxide, a gut microbe–generated phosphatidylcholine metabolite, is associated with autism spectrum disorders. NeuroToxicology. (2020) 76:93–8. 10.1016/j.neuro.2019.10.012 31704102 PMC7385710

[81] Radwan K Wu G Banks-Word K Rosenberger R. An open-label case series of glutathione use for symptomatic management in children with autism Spectrum disorder. Med Sci (Basel). (2023) 11(4):73. 10.3390/medsci11040073 PubMed PMID: 37987328; PubMed Central PMCID: PMC10660524. 37987328 PMC10660524

[82] Ragusa M Santagati M Mirabella F Lauretta G Cirnigliaro M Brex D Potential associations among alteration of salivary miRNAs, Saliva microbiome structure, and cognitive impairments in autistic children. Int J Mol Sci. (2020) 21(17):6203. 10.3390/ijms21176203 32867322 PMC7504581

[83] Reeves KD Figuereo YF Weis VG Hsu FC Engevik MA Krigsman A Mapping the geographical distribution of the mucosa-associated gut microbiome in GI-symptomatic children with autism spectrum disorder. Am J Physiol Gastrointest Liver Physiol. (2024) 327(2):G217–34. 10.1152/ajpgi.00101.2024 PubMed PMID: 38887795; PubMed Central PMCID: PMC11637567. 38887795 PMC11637567

[84] Rose S Bennuri SC Murray KF Buie T Winter H Frye RE. Mitochondrial dysfunction in the gastrointestinal mucosa of children with autism: a blinded case-control study. PLoS One. (2017) 12(10):e0186377. 10.1371/journal.pone.0186377 29028817 PMC5640251

[85] Sherman HT Liu K Kwong K Chan ST Li AC Kong XJ. Carbon monoxide (CO) correlates with symptom severity, autoimmunity, and responses to probiotics treatment in a cohort of children with autism spectrum disorder (ASD): a *post-hoc* analysis of a randomized controlled trial. BMC Psychiatry. (2022) 22(1):536. 10.1186/s12888-022-04151-3 35941573 PMC9358122

[86] Sonbol HM Abdelmawgoud AS El-kady NM Abdelhay ES Abdel Tawab HE. Serum zonulin level in autistic children and its relation to severity of symptoms a case-control study. Sci Rep. (2025) 15(1):27802. 10.1038/s41598-025-11420-0 40738920 PMC12311099

[87] Stewart Campbell A Needham BD Meyer CR Tan J Conrad M Preston GM Safety and target engagement of an oral small-molecule sequestrant in adolescents with autism spectrum disorder: an open-label phase 1b/2a trial. Nat Med. (2022) 28(3):528–34. 10.1038/s41591-022-01683-9 35165451

[88] Su Q Wong OWH Lu W Wan Y Zhang L Xu W Multikingdom and functional gut microbiota markers for autism spectrum disorder. Nat Microbiol. (2024) 9(9):2344–55. 10.1038/s41564-024-01739-1 38977906

[89] Tang Jy Hau Cf Tong Wm Watt RM Yiu CKY Shum Km. Alterations of oral microbiota in young children with autism: unraveling potential biomarkers for early detection. J Dent. (2025) 152:105486. 10.1016/j.jdent.2024.105486 39603332

[90] Teskey G Anagnostou E Mankad D Smile S Roberts W Brian J Intestinal permeability correlates with behavioural severity in very young children with ASD: a preliminary study. J Neuroimmunol. (2021) 357:577607. 10.1016/j.jneuroim.2021.577607 34044209

[91] Turriziani L Ricciardello A Cucinotta F Bellomo F Turturo G Boncoddo M Gut mobilization improves behavioral symptoms and modulates urinary p-cresol in chronically constipated autistic children: a prospective study. Autism Res. (2022) 15(1):56–69. 10.1002/aur.2639 34813183 PMC9299106

[92] Wan Y Zhang L Xu Z Su Q Leung TF Chan D Alterations in fecal virome and bacteriome virome interplay in children with autism spectrum disorder. Cell Rep. Med. (2024) 5(2):101409. 10.1016/j.xcrm.2024.101409 38307030 PMC10897546

[93] Wawer J Chojęta A Wawer GA Gładki M Klotzka A Kociński B The significance of Serum immunoglobulin concentrations in children with autism Spectrum disorders: in search of potential blood biomarkers. Int J Mol Sci. (2025) 26(18):9242. 10.3390/ijms26189242 PubMed PMID: 41009802; PubMed Central PMCID: PMC12470452. 41009802 PMC12470452

[94] Yitik Tonkaz G Esin IS Turan B Uslu H Dursun OB. Determinants of leaky gut and gut Microbiota differences in children with autism Spectrum disorder and their siblings. J Autism Dev Disord. (2023) 53(7):2703–16. 10.1007/s10803-022-05540-z 35441922

[95] Yuan M Wang Q Lu Y Xu P Pan C Zhang W Comparison of gut viral communities between autism spectrum disorder and healthy children. Front Cell Infect Microbiol. (2025) 15:1660970. 10.3389/fcimb.2025.1660970 PubMed PMID: 41163852; PubMed Central PMCID: PMC12558881. 41163852 PMC12558881

[96] Zhai Q Cen S Jiang J Zhao J Zhang H Chen W. Disturbance of trace element and gut microbiota profiles as indicators of autism spectrum disorder: a pilot study of Chinese children. Environ Res. (2019) 171:501–9. 10.1016/j.envres.2019.01.060 30743242

[97] Zhang M Ma W Zhang J He Y Wang J. Analysis of gut microbiota profiles and microbe-disease associations in children with autism spectrum disorders in China. Sci Rep. (2018) 8:13981. 10.1038/s41598-018-32219-2 PubMed PMID: 30228282; PubMed Central PMCID: PMC6143520. 30228282 PMC6143520

[98] Zhang M Chu Y Meng Q Ding R Shi X Wang Z A quasi-paired cohort strategy reveals the impaired detoxifying function of microbes in the gut of autistic children. Sci Adv. (2020) 6(43):eaba3760. 10.1126/sciadv.aba3760 33087359 PMC7577716

[99] Zhong HJ Pan ZY Wei YF Yu Q Wu L Wei H Tongue-coating microbiota as a predictive biomarker of washed microbiota transplantation efficacy in pediatric autism: integration with clinical features. J Transl Med. (2025) 23(1):799. 10.1186/s12967-025-06846-z 40671079 PMC12269187

[100] Zhou J He F Yang F Yang Z Xie Y Zhou S Increased stool immunoglobulin A level in children with autism spectrum disorders. Res Dev Disabil. (2018) 82:90–4. 10.1016/j.ridd.2017.10.009 29102384

[101] Zhu K Xie X Hou F Chen Y Wang H Jiang Q The association between functional variants in long non-coding RNAs and the risk of autism Spectrum disorder was not mediated by gut Microbiota. Mol Neurobiol. (2025) 62(1):412–20. 10.1007/s12035-024-04276-4 38861233

[102] Zurita MF Cárdenas PA Sandoval ME Peña MC Fornasini M Flores N Analysis of gut microbiome, nutrition and immune status in autism spectrum disorder: a case-control study in Ecuador. Gut Microbes. (2020) 11(3):453–64. 10.1080/19490976.2019.1662260 31530087 PMC7524316

[103] Liang Y Xiao Z Ke X Yao P Chen Y Lin L Urinary metabonomic profiling discriminates between children with autism and their healthy siblings. Med Sci Monit. (2020) 26:e926634-1–634-8. 10.12659/MSM.926634 33237888 PMC7702663

[104] Pärtty A Kalliomäki M Wacklin P Salminen S Isolauri E. A possible link between early probiotic intervention and the risk of neuropsychiatric disorders later in childhood: a randomized trial. Pediatr Res. (2015) 77(6):823–8. 10.1038/pr.2015.51 25760553

[105] Özyurt G Öztürk Y Appak YÇ Arslan FD Baran M Karakoyun İ Increased zonulin is associated with hyperactivity and social dysfunctions in children with attention deficit hyperactivity disorder. Compr Psychiatry. (2018) 87:138–42. 10.1016/j.comppsych.2018.10.006 30414552

[106] Stevens AJ Purcell RV Darling KA Eggleston MJF Kennedy MA Rucklidge JJ. Human gut microbiome changes during a 10 week randomised control trial for micronutrient supplementation in children with attention deficit hyperactivity disorder. Sci Rep. (2019) 9:10128. 10.1038/s41598-019-46146-3 PubMed PMID: 31300667; PubMed Central PMCID: PMC6625977. 31300667 PMC6625977

[107] Fernández-López L Molina-Carballo A Cubero-Millán I Checa-Ros A Machado-Casas I Blanca-Jover E Indole tryptophan metabolism and cytokine S100B in children with attention-deficit/hyperactivity disorder: daily fluctuations, responses to methylphenidate, and interrelationship with depressive symptomatology. J Child Adolesc Psychopharmacol. (2020) 30(3):177–88. 10.1089/cap.2019.0072 32048862

[108] Kumperscak HG Gricar A Ülen I Micetic-Turk D. A pilot randomized control trial with the probiotic strain Lactobacillus rhamnosus GG (LGG) in ADHD: children and adolescents report better health-related quality of life. Front Psychiatry. (2020) 11:181. 10.3389/fpsyt.2020.00181 PubMed PMID: 32256407; PubMed Central PMCID: PMC7092625. 32256407 PMC7092625

[109] Pan PY Bölte S. The association between ADHD and physical health: a co-twin control study. Sci Rep. (2020) 10(1):22388. 10.1038/s41598-020-78627-1 33372183 PMC7769983

[110] Jung TH Hwang HJ Han KS. Correlation of attention deficit hyperactivity disorder with gut microbiota according to the dietary intake of Korean elementary school students. PLoS One. (2022) 17(9):e0275520. 10.1371/journal.pone.0275520 PubMed PMID: 36178961; PubMed Central PMCID: PMC9524712. 36178961 PMC9524712

[111] Lee MJ Lai HC Kuo YL Chen VCH. Association between gut Microbiota and emotional-behavioral symptoms in children with attention-deficit/hyperactivity disorder. J Pers Med. (2022) 12(10):1634. 10.3390/jpm12101634 PubMed PMID: 36294773; PubMed Central PMCID: PMC9605220. 36294773 PMC9605220

[112] Perham JC Shaikh NI Lee A Darling KA Rucklidge JJ. Toward ‘element balance’ in ADHD: an exploratory case control study employing hair analysis. Nutr Neurosci. (2022) 25(1):11–21. 10.1080/1028415X.2019.1707395 PubMed PMID: 31900097. 31900097

[113] Yang LL Stiernborg M Skott E Gillberg T Landberg R Giacobini M Lower plasma concentrations of short-chain fatty acids (SCFAs) in patients with ADHD. J Psychiatr Res. (2022) 156:36–43. 10.1016/j.jpsychires.2022.09.042 36228390

[114] Elhossiny RM Elshahawy HH Mohamed HM Abdelmageed RI. Assessment of probiotic strain Lactobacillus acidophilus LB supplementation as adjunctive management of attention-deficit hyperactivity disorder in children and adolescents: a randomized controlled clinical trial. BMC Psychiatry. (2023) 23:823. 10.1186/s12888-023-05324-4 PubMed PMID: 37946220; PubMed Central PMCID: PMC10636814. 37946220 PMC10636814

[115] Robinette LM Hatsu IE Johnstone JM Bruton AM Leung BMY Arnold LE. Treatment response to supplemental nutrients for ADHD is independent of diet quality: the MADDY study RCT. Nutr Neurosci. (2024) 27(4):319–28. 10.1080/1028415X.2023.2191415 36989335 PMC10539486

[116] Ast HK Hammer M Zhang S Bruton A Hatsu IE Leung B Gut microbiome changes with micronutrient supplementation in children with attention–deficit/hyperactivity disorder: the MADDY study. Gut Microbes. (2025) 17(1):2463570. 10.1080/19490976.2025.2463570 PubMed PMID: 39963956; PubMed Central PMCID: PMC11845018. 39963956 PMC11845018

[117] Steckler R Magzal F Kokot M Walkowiak J Tamir S. Disrupted gut harmony in attention-deficit/hyperactivity disorder: dysbiosis and decreased short-chain fatty acids. Brain Behav Immun Health. (2024) 40:100829. 10.1016/j.bbih.2024.100829 PubMed PMID: 39184374; PubMed Central PMCID: PMC11342906. 39184374 PMC11342906

[118] Krajewski K. Heavy metals, noradrenaline/Adrenaline ratio, and microbiome-associated hormone precursor metabolites: biomarkers for social behaviour, ADHD symptoms, and executive function in children. Sci Rep. (2025) 15(1):19006. 10.1038/s41598-025-00680-5 40447636 PMC12125380

[119] Lawrence K Fibert P Toribio-Mateas M Gregory AM Hobbs J Quadt F Effects of kefir on symptoms, sleep, and gut microbiota in children with ADHD: a randomised controlled trial. BMC Psychiatry. (2025) 25:1117. 10.1186/s12888-025-07568-8 PubMed PMID: 41286799; PubMed Central PMCID: PMC12642372. 41286799 PMC12642372

[120] Niu Q Wang W Liang Y Fang S Wan L Yang G. Efficacy and safety of a probiotic mixture containing Bifidobacterium animalis subsp. lactis BLa80 and lacticaseibacillus rhamnosus LRa05 in children with attention-deficit/hyperactivity disorder. Mol Nutr Food Res. (2025) 69(22):e70234. 10.1002/mnfr.70234 40898729

[121] Schopler E Reichler RJ DeVellis RF Daly K. Toward objective classification of childhood autism: childhood autism rating scale (CARS). J Autism Dev Disord. (1980) 10(1):91–103. 10.1007/BF02408436 6927682

[122] Aman MG Singh NN Stewart AW Field CJ. The aberrant behavior checklist: a behavior rating scale for the assessment of treatment effects. Am J Ment Defic. (1985) 89(5):485–91. PubMed PMID: 3993694. 3993694

[123] Constantino JN Gruber CP. Social Responsiveness Scale (SRS) Manual. Los Angeles: Western Psychological Services (2005).

[124] Njotto LL Simin J Fornes R Odsbu I Mussche I Callens S Maternal and early-life exposure to antibiotics and the risk of autism and attention-deficit hyperactivity disorder in childhood: a Swedish population-based cohort study. Drug Saf. (2023) 46(5):467–78. 10.1007/s40264-023-01297-1 37087706 PMC10164008

[125] Chen Y Xueying Z Jiaqu C Qiyi C Huanlong Q Ning L FTACMT Study protocol: a multicentre, double-blind, randomised, placebo-controlled trial of faecal microbiota transplantation for autism spectrum disorder. BMJ Open. (2022) 12(1):e051613. 10.1136/bmjopen-2021-051613 35105621 PMC8804636

[126] Korteniemi J Karlsson L Aatsinki A. Systematic review: autism spectrum disorder and the gut microbiota. Acta Psychiatr Scand. (2023) 148(3):242–54. 10.1111/acps.13587 37395517

[127] Chen Z Shi K Liu X Dai Y Liu Y Zhang L Gut microbial profile is associated with the severity of social impairment and IQ performance in children with autism Spectrum disorder. Front Psychiatry. (2021) 12:789864. 10.3389/fpsyt.2021.789864 PubMed PMID: 34975585; PubMed Central PMCID: PMC8718873. 34975585 PMC8718873

[128] Levy Schwartz M Magzal F Yehuda I Tamir S. Exploring the impact of probiotics on adult ADHD management through a double-blind RCT. Sci Rep. (2024) 14:26830. 10.1038/s41598-024-73874-y PubMed PMID: 39500949; PubMed Central PMCID: PMC11538393. 39500949 PMC11538393

[129] Sun W Ma L Feng X Fan Y Cai Y Li X. Efficacy of gut microbiota-based therapy for autism Spectrum disorder and attention deficit hyperactivity disorder: a systematic review and meta-analysis. Psychol Health Med. (2025) 0(0):1–25. 10.1080/13548506.2025.2565181 PubMed PMID: 41037658. 41037658

[130] Grant MJ Booth A. A typology of reviews: an analysis of 14 review types and associated methodologies. Health Info Libr J. (2009) 26:91–108. 10.1111/j.1471-1842.2009.00848.x 19490148

