# Supplementary material for: The microbiota–gut–brain axis as a modulator of symptom expression in autism spectrum disorder, with exploratory insights into ADHD: evidence from a structured narrative review on paediatric population
Source: Front Child Adolesc Psychiatry. 2026 May 28;5:1835043. doi: 10.3389/frcha.2026.1835043 (PMC13253956; doi:10.3389/frcha.2026.1835043)
Supplement: Supplementary file 1 [file Table1.pdf]

Citation (Author, year)	Reference	Study design	Age range	Sample size	DSM and/or ICD diagnostical features	Methodological approaches across studies	Autism-related measures (diagnosis, severity, and symptom domains)	Behavioural scale	Clinical associations involving ASD symptoms, gastrointestinal features, and diet	Interventional studies and clinical outcomes	Main findings
Anwar et al. (2016)	23	C-CS	5-12	48 (ASD 27; CG 21)	DSM-5	Plasma and urine collection; HPLC with fluorimetric detection (thiamine, TMP, TPP); LC-MS/MS for plasma protein oxidative damage markers (dityrosine, N-formylkynurenine, 3-nitrotyrosine); CARS; ADOS	ADOS	CARS	Plasma thiamine and TMP levels and ASD VS CG: NSA; Plasma TPP ↓ ASD vs CG; Plasma protein dityrosine ↑ ASD vs CG; thiamine metabolites and ASD clinical severity: NSA; Diet: NA	NA	Plasma TPP decreased and plasma protein dityrosine increased in ASD vs controls
Fiorentino et al. (2016)	39	CC-POSTM	3-46	ASD: 8 brain, 12 duodenum; CG: 15 brain, 9 duodenum	NR	Duodenal biopsy; gene and protein expression of tight-junction and inflammatory markers; qPCR; Western blot	NA	NR	Altered intestinal and BBB markers in ASD; SSR: NA; GI features: NA; Diet: NR	NA	Altered blood–brain barrier and intestinal epithelial barrier markers observed in ASD
Fulceri et al. (2016)	4	C-CS	2–6	230 (ASD 115; CG 115)	DSM-IV-TR	GI symptom assessment; behavioral evaluation	NR	CBCL	GI symptoms assoc with greater behavioral problems in ASD; Diet: NA	NA	GI symptom presence and severity associated with increased behavioral difficulties in preschool children with ASD
Gabriele et al. (2016)	40	C-CS	1-20	53 ASD	DSM-IV-TR	BSC; urinary p-cresol; intestinal transit time assessment	NR	NR	Slow intestinal transit assoc with urinary p-cresol ↑; SSR: NA; Diet: NA	NA	Elevated urinary p-cresol associated with slower intestinal transit in ASD
Gevi et al. (2016)	42	C-CS	2-7	60 (ASD 30; CG 30)	DSM-IV	BSC; urinary metabolomics (HILIC–UHPLC–MS); pathway-based statistical analysis	ADOS; ADI-R;	NR	SSR: NA; GI features: NA; Diet: NA	NA	Altered tryptophan and purine metabolism and increased microbiome-derived metabolites observed in ASD
Inoue et al. (2016)	45	C-CS	3-5	12 (ASD 6; CG 6)	DSM-5	FSC; 16S rRNA GS; PBMC gene expression microarray; correlation analysis	PARS; M-CHAT	NR	GMC correlated with immune-related gene expression; SSR: NA; GI features: NA; Diet: NA	NA	Altered gut microbiota composition in ASD infants associated with interferon-related immune gene expression
Jory et al. (2016)	47	C-CS	2-4	26 (ASD 11; CG 15)	DSM (not versione specified)	BSC; plasma fatty acid profiling	NA	NA	No GI assessment; dietary intake requested (5-day food diary) but insufficient compliance; no diet analysis performed	NA	ASD group showed ↓ RBC DHA, EPA, AA, and ↓ ω-3/ω-6 ratio; ↓ serum DHA, AA, LA. Authors propose potential link between PUFA abnormalities, cobalamin metabolism, gut microbiota, and propionic acid in ASD pathophysiology
Kantarcioglu et al. (2016)	54	CC	9 m -18 y	1958 (ASD 1555; CG 403)	NR	FSC; stool culture; yeast species identification; microbiological assays	NR	NR	Yeast species prevalence ↑ in ASD vs CG; SSR: NA; GI features: NA; Diet: NA	NA	Yeast species isolated more frequently from stool samples of children with ASD than controls
Marler et al. (2016)	70	CS	6-18	82 ASD (NO CG)	DSM-IV	BSC; whole blood serotonin (HPLC); IL-6 (ELISA); QPGS (Rome III); ADOS; RBS-R; SensOR; ABC	ADOS	ABC; SensOR;RBS-R	↑ whole blood 5-HT ↔ ↑ GSS (QPGS lower GI score); Ø association with FC diagnosis; Ø association with behavioral scales (ABC, RBS-R, SensOR); Ø association with IL-6; Diet: NA	NA	Whole blood serotonin levels weakly correlated with lower GI symptom severity in ASD
Esnafoglu et al. (2017)	38	C-CS	4–12	65 (ASD 32; CG 33)	DSM-5	BSC; serum zonulin (ELISA); autism severity assessment (CARS); gastrointestinal symptom questionnaire	CARS	NR	Zonulin ↑ assoc with SSR; Zonulin ↑ assoc with GI symptoms; Diet: NA	NA	Serum zonulin levels increased in ASD and associated with ASD symptom severity and GI symptoms
Kang et al. (2017)	51	PILOT INT-OL-PP	7–16	38 (ASD 18; CG 20)	NR	Antibiotics; bowel cleanse; fecal microbiota transfer (MTT); GI symptom scales; ASD symptom scales; FSC; microbiota profiling	ADI-R	NA	GI symptoms ↓ and ASD symptoms ↓ post-MTT; longitudinal pre–post assessment	Microbiota Transfer Therapy (FMT/MTT)	Microbiota Transfer Therapy associated with improvements in GI symptoms and ASD-related behaviors
Liu et al. (2017)	66	PILOT INT-OL-PP	1-8	64 ASD (NO CG)	DSM-5	FSC; 16S rRNA GS; BTA; GMC; BSC; serum retinol; vitamin A supplementation	ABC; CARS	ABC; SRS	SSR ↓ post-supplementation vs baseline; Baseline GSS–SSR: Ø; Diet: NA	Vitamin A supplementation; outcomes: GMC changes; SSR (CARS) ↓	Vitamin A supplementation altered gut microbiota composition and reduced ASD symptom severity
Lussu et al. (2017)	69	CC	ASD 4-16; Siblings 4-17	42(ASD 21; CG 21)	NR	BSC; urinary metabolomics (1H-NMR); multivariate analysis	ADOS	NR	Urinary metabolites differed ASD vs SIB; GSS–urinary metabolites: Ø; Diet: NA	NA	Distinct urinary ^{1}H-NMR metabolomic profile observed in ASD vs siblings and controls
Marler et al. (2017)	71	CS	6-18	108 ASD (NO CG)	DSM-IV-TR	Questionnaire on Pediatric Gastrointestinal Symptoms–Rome III (QPGS-RIII); Repetitive Behavior Scale–Revised (RBS-R); SensOR; ABC; ADOS	ADOS	ABC; SensOR;RBS-R	Whole blood serotonin–GSS (QPGS): ↑ (weak); Whole blood serotonin–SSR: Ø; Diet: NA	NA	Functional constipation associated with increased rigid-compulsive behaviors in ASD
Rose et al. (2017)	84	CC	5-17	30 (ASD GI 10; CHRON'S 10; NS GI CONTROL 10)	DSM-IV	GI mucosal biopsy sampling (rectum, cecum); mitochondrial function assays; citrate synthase; Western blot (ETC complexes I–V); ADI-R; ADOS	ADOS; ADI-R	NA	Mitochondrial complex I activity ↑ ASD vs CG (GI mucosa); ETC protein content (complexes I, III, IV, V) ↑ ASD vs CG (cecum); Citrate synthase: NSA; GSS: NA; Diet: NA	NA	Increased mitochondrial complex I activity and ETC protein content in GI mucosa of children with ASD
Bent et al. (2018)	26	PILOT INT-OL-PP	7-21	15 (ASD 15; NO CG)	DSM-IV	Urine; open-label sulforaphane supplementation (12 weeks); urinary metabolomics (UHPLC/MS by Metabolon); fasting urine samples pre/post; ABC; SRS; Pearson correlation analysis between metabolite changes and symptom changes	NR	ABC; SRS	Changes in urinary metabolites ↓ SSR; ↑ microbiome-associated metabolites ↓ SSR; Diet: NA	Sulforaphane for 12 weeks; SRS total score ↓ (significant); ABC total score ↓ (NSA); responders (≥4-point ABC improvement) showed greater ABC and SRS reductions	Urinary metabolomic changes correlated with clinical improvements following sulforaphane treatment
Góra et al. (2018)	44	C-CS	3-18	70 (ASD 29; Obese CG 24; Healthy CG 17)	NR	FSC; isolation of Clostridium perfringens; toxin gene profiling (PCR); cytotoxicity assays	NA	NA	CP toxigenic strains ↑ in ASD vs CG; GI features: NA; Diet: NA	NA	Higher prevalence of toxigenic Clostridium perfringens strains in ASD vs controls

Citation (Author, year)	Reference	Study design	Age range	Sample size	DSM and/or ICD diagnostical features	Methodological approaches across studies	Autism-related measures (diagnosis, severity, and symptom domains)	Behavioural scale	Clinical associations involving ASD symptoms, gastrointestinal features, and diet	Interventional studies and clinical outcomes	Main findings
Józefczuk et al. (2018)	48	C-CS	2.5-17.9	123 (77 ASD; 46 CG)	NR	BSC; serum immunoassays for gluten-related antibodies (AGA IgA/IgG, anti-TG2, anti-DGP, anti-TG6); zonulin and I-FABP (ELISA); correlation and regression analyses	NR	NR	Gluten-related antibodies ↑ in ASD subgroup; Antibody positivity–intestinal permeability markers: Ø; Age-related increase in antibody prevalence; Diet at inclusion: no GFCF	NA	Gluten-related antibodies detected in a subset of children with ASD
Kang et al. (2018)	52	C-CS	4-17	44 (ASD 23; CG 21)	NR	FSC; 1H-NMR fecal metabolomics; 16S rRNA GS; GI Severity Index (6-GSI); ATEC; PDD-BI; Fisher Discriminant Analysis	ATEC; PDD-BI	NA	Fecal metabolites assoc with GI severity and ASD symptoms; p-cresol levels correlated with age (ASD only); Diet: NA	NA	Higher fecal isopropanol levels and altered fecal metabolite profiles observed in ASD vs controls
Qiao et al. (2018)	79	C-CS	7-14	59 (ASD 32; CG 27); oral samples 111	DSM-5/ICD10	Oral sample collection; 16S rRNA GS (V3–V4); BTA; MC; ABC; oral health indices (DMFT/DMFS, PLI, GI, BOP, PD)	ABC	NA	Oral MC diversity ↓ ASD vs CG; Proteobacteria, Streptococcus, Haemophilus ↑ ASD vs CG; Prevotella ↓ ASD vs CG; Haemophilus–SSR (ABC): ↑; Rothia–SSR (ABC): ↑; GSS: NA; Diet: NA	NA	Distinct salivary and dental plaque microbiota profiles associated with autism severity
Rose et al. (2018)	5	C-CS	3-12	103 (ASD GI 20; ASDNoGI 26; CG GI 6; CGNoGI 35)	DSM-IV	BSC; serum cytokines (multiplex immunoassay); FSC; 16S rRNA GS; BTA; GMC; ADI-R; ADOS	ADOS; ADI-R	ABC; SCQ	Pro-inflammatory cytokines ↑ ASD vs CG; GMC differed ASD vs CG; Cytokines–SSR: Ø; GSS: NA; Diet: NA	NA	Altered immune profiles and gut microbiota composition observed in ASD
Zhang et al. (2018)	94	C-CS	3–8	41 (ASD 35; CG 6)	DSM-5	FSC; 16S rRNA GS (V3–V4); BTA; GMC; alpha/beta diversity; microbe–disease network analysis (HMDAD/HMDN)	NR	NR	B/F ratio ↑ ASD vs CG; Sutterella/Odoribacter/Butyricimonas ↑ ASD vs CG; Veillonella/Streptococcus ↓ ASD vs CG; GSS: NA; Diet: NA	NA	Altered gut microbiota composition potentially related to ASD pathogenesis
Zhou et al. (2018)	100	C-CS	1-6	74 (ASD 43; CG 31)	DSM-5	FSC; fecal IgA (ELISA); GSS	NR	NR	Fecal IgA ↑ ASD vs CG; Fecal IgA–GSS: Ø; Diet: NA	NA	Higher fecal IgA levels observed in ASD; no association with GI symptom severity
Belardo et al. (2019)	25	CC	3-8	120 (ASD 60; CG 60)	DSM (not versione specified)	Urine; metabolomics (UHPLC–MS); quantification of vitamin B6-, B9-, and B12-related metabolites; assessment of methylation and trans-sulfuration pathways; oxidative stress markers	NA	NA	Plasma vitamin B6, B9, B12 levels ↓ in ASD vs controls; Vitamin levels-SSR: NSA; Gastrointestinal features: NA; Diet:NA	NA	Lower circulating vitamin B6, B9, and B12 levels in ASD
Kang et al. (2019)	53	INT-OL-LONG	7–17	18 ASD (NO CG)	DSM-IV	Long-term follow-up after MTT; ASD symptom scales; GI symptom assessment; FSC; microbiota profiling	CARS; SRS	NA	Sustained GI symptoms ↓ and ASD symptoms ↓ up to 2 years post-MTT	Microbiota Transfer Therapy (follow-up)	Improvements in GI and ASD symptoms persisted up to two years post-treatment
Kong et al. (2019)	60	C-CS	7-25	39 (ASD 20; CG 19)	DSM-5	FSC; oral sample collection; 16S rRNA GS; BTA; GMC	NR	NR	GMC differed ASD vs CG (oral and gut microbiota); SSR: NA; GSS: NA; Diet: NA	NA	Distinct oral and gut microbiota profiles differentiated ASD from controls
Li et al. (2019)	65	C-CS	ASD 2-7; CG 2-10; ASD Mothers 26-38; CG Mothers 27-42	178 (ASD 59; CG 30; ASD M 59; CG 30)	DSM-5	FSC; 16S rRNA GS (V3–V4); BTA; GMC; alpha diversity (Chao1, Shannon); beta diversity (UniFrac PCA); LEfSe; correlation analyses; ROC analysis	ABC	NA	GMC differed ASD vs CG; Alpha diversity (richness) ↑ ASD vs CG; Proteobacteria ↑ ASD vs CG; Clostridium ↑, Acinetobacter ↑ ASD vs CG; Prevotella ↓ ASD vs CG; GMC differed ASD mothers vs CG mothers; Mother–child GMC profiles correlated; GSS: NA; Diet: NA	NA	Distinct gut microbiota profiles in ASD, with taxa associated with ASD symptom severity
Liu et al. (2019)	67	C-CS	2.5-18	50 (ASD 30; CG 20)	DSM-5/ICD10	FSC; 16S rRNA GS; BTA; GMC; fecal SCFA analysis (HPLC); GSS; correlation analyses	NA	NA	GMC differed ASD vs CG; Fecal acetate ↓ ASD vs CG; Fecal butyrate ↓ ASD vs CG; Valeric acid ↑ ASD vs CG; Constipation ↑ ASD vs CG; Diet: NA	NA	Altered gut microbiota composition, reduced acetate and butyrate, increased valeric acid, and higher constipation prevalence in ASD
Plaza-Díaz et al. (2019)	78	C-CS	2–6	105 (30 ASD ANMR; 18 ASD AMR; CG 57)	DSM-5/ICD10	FSC; 16S rRNA GS; BTA; GMC; fecal SCFA analysis (gas chromatography); CARS	ADI-R	NA	GMC differed ANMR vs AMR; SCFA profiles differed ANMR vs AMR; GMC–SSR (CARS): Ø; GSS: NA; Diet: NA	NA	Distinct gut microbiota and SCFA profiles in ASD with mental regression vs ASD without regression
Zhai et al. (2019)	96	C-CS	3-7	136 (ASD 78; CG 58)	DSM-IV/ICD10	Hair trace element analysis; fecal sample collection; 16S rRNA GS; redundancy analysis (RDA); random forest modeling	ATEC	ATEC	Hair trace element analysis; FSC; 16S rRNA GS; BTA; GMC; RDA; random forest modeling	NA	Hair trace element alterations and gut microbiota differences observed in ASD
Ahmed et al. (2020)	20	C-CS	2-18	131 (ASD 41; SIBLINGS 45; CG 45)	DSM-5	FSC; fecal DNA extraction; qPCR (16S rRNA); relative abundance analysis; F/B and P/B ratios; alpha diversity (Shannon); similarity (Bray–Curtis); Spearman correlations	CARS	NR	Microbiota–ASD severity (CARS): NSA; Microbiota–GI severity (6-GSI): NSA; Diet: NSA	NA	Gut microbiota differed in ASD (and siblings) vs controls (↑ Bacteroides, Ruminococcus; ↓ F/B and P/B ratios); no associations with ASD symptom severity
Alshammari et al. (2020)	22	C-CS	3-12	114 (ASD 57; CG 57)	NR	FSC; anaerobic culture; VITEK 2 identification; PCR detection of Cpa and Cpb2 toxin genes; Cpb2 sequencing; antimicrobial susceptibility testing (E-test); subgroup analysis by GI symptoms	NR	NR	CP incidence ↑ in ASD vs CG; CP incidence ↑ ASD+GI vs ASD-GI; Cpb2 toxin gene prevalence ↑ ASD+GI vs ASD-GI; Diet: NA	NA	Clostridium perfringens prevalence increased in ASD, particularly in ASD with GI symptoms; increased antibiotic resistance in ASD isolates
Averina et al. (2020)	24	CC	3-5	57 (ASD 36; CG 21)	NR	FSC; shotgun metagenomic sequencing; taxonomic profiling (MetaPhlAn2); bioinformatic identification of neurometabolic pathways	NR	NR	NR	NA	Reduced abundance of microbial genes involved in neurometabolic pathways in ASD
Chen et al. (2020)	31	C-CS	2.5-8.2	123 (ASD 76; CG 47)	NR	FSC; 16S rRNA gene sequencing; BTA; developmental assessment scales; social behavior scales; mother–child paired analyses; correlation analyses	CARS; ADOS-2; ADI-R	NR	Shared mother–child taxa ↑ corr with developmental level; Shared taxa ↓ corr with social deficits; GI features: NA; Diet: NA	NA	Shared mother–child gut bacterial taxa associated with higher developmental level and lower social deficits in children with ASD

Citation (Author, year)	Reference	Study design	Age range	Sample size	DSM and/or ICD diagnostical features	Methodological approaches across studies	Autism-related measures (diagnosis, severity, and symptom domains)	Behavioural scale	Clinical associations involving ASD symptoms, gastrointestinal features, and diet	Interventional studies and clinical outcomes	Main findings
Gevi et al. (2020)	43	C-CS	3-8	80 (ASD 40; CG 40)	DSM-5	BSC; urinary metabolomics (UHPLC–MS); targeted analysis of neurotransmitter-related metabolites; multivariate analysis (PCA, OPLS-DA); pathway analysis	NR	NR	NA	NA	Altered urinary neurotransmitter-related metabolites in ASD vs controls
Kandeel et al. (2020)	50	C-CS	2–8	60 (ASD 30; CG 30)	DSM-5	FSC; qPCR (16S rRNA); stool analysis; CARS; ADI-R; anthropometric measures	ADI-R; CARS;	NR	Clostridium spp. abundance ↑ in ASD vs CG; Clostridium counts–SSR: Ø; Clostridium counts–anthropometric measures: Ø	NA	Higher stool abundance of several Clostridium species in ASD vs controls
Kovtun et al. (2020)	62	C-CS	2-9	50 (ASD 30; CG 20)	NR	FSC; shotgun metagenomic GS; BTA; GMC; ARG annotation (ResFinder); group comparisons	CARS	NR	ARG abundance ↑ ASD vs CG; ARG–SSR: Ø; GSS: NA; Diet: NA	NA	Increased abundance of antibiotic resistance genes in ASD gut microbiota
Laue et al. (2020)	64	COH-P	6w - 3y	273 (sequencing samples: 6 weeks 166, 1 year 158, 2 years 129, 3 years 140; metagenomics subset at 6 weeks 101 and 1 year 103)	NR	FSC; 16S rRNA GS; metagenomic GS; BTA; GMC; functional pathway analysis; parent-reported social behavior scales	SRS-2	NR	GMC/function–social behavior scores: ↑; GSS: NA; Diet: NA	NA	Infant gut microbiome composition associated with later childhood social behavior scores
Liang et al. (2020)	103	C-CS	3-12	44 (ASD 22; CG 22)	DSM-IV	BSC; urinary metabolomics (GC–MS / LC–MS); multivariate analysis	ABC; CARS	NR	Urinary metabolomic profiles differed ASD vs CG; SSR: NA; GSS: NA; Diet: NA	NA	Urinary metabolomic profiles discriminated ASD children from healthy siblings
Quan et al. (2020)	80	C-CS	3-8	328 (ASD164; CG 164)	DSM-5	BSC; plasma metabolomics (LC–MS/MS); TMAO; choline; betaine; logistic regression; ROC analysis	CARS	NR	Plasma TMAO ↑ ASD vs CG; Plasma TMAO–SSR (CARS): ↑; Plasma TMAO ↑ severe vs mild/moderate ASD; GSS: NA; Diet: NA	NA	Elevated plasma TMAO levels associated with ASD diagnosis and ASD symptom severity
Ragusa et al. (2020)	82	C-CS	6-14	80 (ASD 40; CG 40)	DSM-5	Oral sample collection; salivary miRNA profiling; 16S rRNA GS; BTA; MC; cognitive assessment scales	ADOS; ADI-R	NNR	Salivary miRNAs differed ASD vs CG; Salivary MC differed ASD vs CG; Salivary miRNAs–cognitive scores: ↑; GSS: NA; Diet: NA	NA	Distinct salivary miRNA and saliva microbiome profiles associated with cognitive measures in ASD
Tomova et al. (2020)	13	C-CS	ASD 4-8.5; CG 2.8-9.15	62 (ASD 46; CG 16)	DSM-5	Dietary assessment (FFQ, dietary records); FSC; 16S rRNA GS; BTA; GMC; GSS	ADOS; ADI-R	NR	Food selectivity ↑ ASD vs CG; GMC diversity ↓ ASD vs CG; Specific taxa differed selective vs non-selective ASD; Diet–SSR: Ø; GSS: NA	NA	Food selectivity associated with reduced gut microbial diversity and altered microbiota composition in ASD
Zhang et al. (2020)	98	C-CS	3-8	79 (ASD 39; CG 40)	DSM-5	FSC; shotgun metagenomic GS; BTA; GMC; quasi-paired cohort analysis; urinary organic acid quantification; random forest modeling	ADI-R; ADOS-2; CARS; ABC	NR	Microbial detoxification pathways ↓ ASD vs CG; Detoxification dysfunction score–SSR: ↑; GSS: NA; Diet: NA	NA	Impaired microbial detoxification pathways associated with increased ASD symptom severity
Zurita et al. (2020)	102	CC	5-12	60 (ASD 25; CG 35)	NR	ADI-R; SCQ; anthropometry; 24h dietary recall; FFQ; BSC; serum cytokines (TGF-β1, IL-6, IFN-γ, MCP-1); anti-tTG IgA/IgG (ELISA); FSC; 16S rRNA GS (V4); BTA; alpha diversity (Shannon, Faith's PD); beta diversity (Bray–Curtis, UniFrac)	ADI-R	SCQ	Constipation ↑ ASD vs CG; Food aversion/intolerance ↑ ASD vs CG; TGF-β1 ↑ ASD vs CG; GMC alpha-diversity ↑ ASD vs CG; GMC beta-diversity ≠ ASD vs CG; GMC–diet: Ø; Diet–SSR: Ø	NA	Increased GI symptoms, higher serum TGF-β1 levels, and altered gut microbiota composition observed in ASD
Jendraszak et al. (2021)	46	C-CS	3-9	73 (ASD: 33; healthy CG: 16; allergy CG: 24)	NR	FSC; commercial stool microbiota test; relative abundance comparison	DI-R; ADOS-2; CARS; M-CHAT/Q-CHAT/CAST	NR	GMC differed between ASD and CG; SSR: NA; GI features: NA; Diet: NA	NA	Commercial microbiota testing showed compositional differences between ASD and control children
Khalil et al. (2021)	57	C-CS	ASD 3-10; SB 0.5 - 12 ; CG 2-12	ASD: 58; siblings: 45; unrelated controls: 45	DSM-5	FSC; qPCR (16S rRNA); PCR toxin A/B genes; CARS; SSP; GSS	CARS; SSP	NR	Clostridium difficile: NSA ASD vs siblings vs CG; C. difficile–SSR: Ø; C. difficile–GSS: Ø; Diet: NA	NA	No differences in Clostridium difficile prevalence, abundance, or toxin production between ASD, siblings, and controls
Kong et al. (2021)	61	C-CS	7-25	39 (ASD 20; CG 19)	DSM-5	FSC; 16S rRNA GS; BTA; GMC; autonomic function assessment (EDA, HR, HRV, IBI, BVP); ATEC; correlation analyses	ATEC	NR	GMC alpha diversity ↓ with SSR; Autonomic indices–SSR: ↓/altered; GSS: NA; Diet: NA	NA	Lower gut microbiome alpha diversity associated with greater ASD symptom severity
Liu et al. (2021)	68	C-CS	NR (children)	52 (ASD 26; CG 26)	DSM-5	FSC; shotgun metagenomic GS; BTA; GMC; whole-exome sequencing; fecal metabolomics (UHPLC–MS); BSC; serum cytokines; regression analyses; causal inference test	NA	NR	ASD-associated SNVs–GMC: ↑; GMC–SSR: ↑; Microbial metabolic pathways altered ASD vs CG; Fecal neurotransmitter-related metabolites ↑ ASD vs CG; Proinflammatory cytokines ↑ ASD vs CG; GSS: NA; Diet: NA	NA	Functional ASD-associated SNVs were correlated with gut microbiome diversity, specific bacterial taxa, microbial metabolic pathways, fecal neurotransmitter-related metabolites, and elevated proinflammatory cytokines compared with controls
Palkova et al. (2021)	76	CS	2.8-9.2	10 ASD (NO CG)	DSM-5/ICD10	FSC; 16S rRNA GS; MiSeq; primer set comparison (V1–V2 vs V3–V4 vs V4–V5); QIIME2; DADA2; OTU/ASV-based MC; alpha diversity; beta diversity; BTA; SILVA	ADOS-2; ADI-R;	NR	NA	NA	MC ≠ depending on 16S rRNA primer set. Significant differences in alpha diversity, beta diversity, and relative abundance of major taxa across primer regions. Primer choice identified as a major source of methodological heterogeneity in ASD GMC studies, potentially contributing to inconsistent results across the literature.
Teskey et al. (2021)	90	CS	2-5	32 ASD (NO CG)	DSM-IV-TR	BSC; serum zonulin (ELISA); serum I-FABP (ELISA); CARS; GSS	ADI-R; ADOS	NR	Serum zonulin–SSR (CARS): ↑; Serum I-FABP–SSR (CARS): ↑; Intestinal permeability markers–GSS: Ø; Diet: NA	NA	Higher intestinal permeability markers associated with greater ASD symptom severity in very young children

Citation (Author, year)	Reference	Study design	Age range	Sample size	DSM and/or ICD diagnostical features	Methodological approaches across studies	Autism-related measures (diagnosis, severity, and symptom domains)	Behavioural scale	Clinical associations involving ASD symptoms, gastrointestinal features, and diet	Interventional studies and clinical outcomes	Main findings
Yap et al. (2021)	12	C-CS	3-18	247 (ASD 99; CG 148)	NR	Dietary preference assessment; FSC; 16S rRNA GS; BTA; GMC	ADOS-2; ADI-R; SRS-2; SCQ	NR	Dietary selectivity mediated GMC–ASD status association; GMC differences ASD vs CG attenuated after diet adjustment; GSS: NA; Diet: mediated effect	NA	Dietary preferences partially mediated the association between gut microbiota composition and ASD
Alookaran et al. (2022)	21	C-CS	4-16	50 (ASD+GI 20; ASD–GI 10; TD controls 20)	DSM-5	FSC; ITS2 sequencing (mycobiome); qPCR (fungal load); alpha diversity (OTUs, Shannon); beta diversity (Bray–Curtis PCA); Candida spp. detection; fecal calprotectin (ELISA); serum dectin-1 (ELISA); group comparisons; correlation analyses	ADI-R	SRS-2; SCQ	Candida–ASD severity: NSA; Candida–GI severity: NSA; Fungal measures–calprotectin: NSA; Diet: NA	NA	No differences in fecal fungal diversity or abundance between ASD (with or without GI symptoms) and TD children; Candida spp. not associated with behavioral severity, GI symptoms, gut inflammation, or antifungal immunity
Billeci et al. (2022)	28	RCT - secondary analysis	3–6	63 ASD; EEG 46 (Probiotics 26; Placebo 20)	DSM-5	Probiotic supplementation (Vivomixx®, 6 months); high-density EEG (128 channels); resting-state power, coherence, asymmetry analyses; ADOS-2; CARS; RBS-R; GSI; cytokines (TNF-α, IL-6, CCL2); Pearson correlations between EEG, clinical and inflammatory measures	ADOS-2; CARS; ADI-R; RBS-R; SCQ	CBCL	Frontopolar beta/gamma power ↓ and coherence ↑ in probiotics vs placebo; EEG changes correlated with ↓ repetitive behaviors (RBS-R); frontopolar coherence negatively correlated with TNF-α; GI symptoms (GSI): NSA; Diet: NA	Probiotic supplementation for 6 months; outcomes: EEG power, coherence, asymmetry, behavioral and inflammatory measures	Probiotic supplementation induced EEG changes correlated with behavioral and inflammatory measures in preschool children with ASD
Chiappori et al. (2022)	33	C-CS	ASD 6-17 ; CG 10-20	12 (ASD 6; CG 6)	DSM-5	FSC; GMC profiling (16S rRNA GS; BTA); fecal small ncRNA profiling (miRNA, piRNA); integrative multi-omics analysis	NR	NA	GMC–ncRNA integrated profiles discriminate ASD vs CG; SSR: NA; GI features: NA; Diet: NA	NA	Combined fecal microbiota and small ncRNA profiles discriminated ASD from controls
Daneberga et al. (2022)	35	CS	2–12	44(NO CG)	ICD10	Urine; organic acid analysis (GC–MS); FSC; GMC profiling; dietary questionnaire (parental interview)	ADOS	NA	Urinary metabolites–SSR: Ø; GMC–SSR: Ø; Diet recorded: NSA; GI features: NA	NA	No clear urinary metabolite abnormalities; p-cresol showed high interindividual variability; HPHPA not confirmed
Karagözlü et al. (2022)	55	C-CS	3–18	111 (ASD 56; CG 55)	DSM-5	BSC; serum zonulin (ELISA); CARS; GI symptom questionnaire	CARS	NA	Serum zonulin levels correlated with autism severity and gastrointestinal symptoms; diet not assessed	NA	Higher serum zonulin levels associated with greater ASD symptom severity and GI symptom burden
Kartalc et al. (2022)	56	C-CS	3-12	70 (ASD 35; CG 35)	DSM-5	BSC; serum zonulin; anti-gliadin IgA/IgG; cytokines (IL-6, TNF-α, TGF-β); neuronal markers (S100B, NSE); ELISA	NR	NA	Serum zonulin ↓ ASD vs CG; GI symptoms (infancy/current): ≠; AGA IgA: ≠; AGA IgG: ≠; IL-6 ↑ ASD vs CG; TGF-β ↑ ASD vs CG; TNF-α: ≠; S100B: ≠; NSE: ≠; GSS: ≠; Diet: NA	NA	Lower serum zonulin levels and higher IL-6 and TGF-β levels observed in ASD vs controls
Kim et al. (2022)	58	COH-P	NR (children)	568 (microbioma data 170; postnatal expousure analyses 132)	NR	FSC; 16S rRNA GS (V3–V4); BTA; GMC; alpha diversity (Chao, Shannon, Inverted Simpson); multivariable regression; mediation analysis	SCQ (autistic traits; no clinical diagnosis)	NR	Prenatal PM10, NO_2 ↑ → autistic traits (SCQ); Proteobacteria ↑ → autistic traits (SCQ); Proteobacteria partially mediate PM10/NO_2–autistic traits association; GSS: NA; Diet: NA	NA	Prenatal air pollution exposure associated with increased autistic traits; gut microbiota composition partially mediated the association
Meguid et al. (2022)	73	INT-OL-PP	2–5	40 ASD	DSM-5	FSC; qPCR (16S rRNA); Bifidobacterium spp.; Lactobacillus spp.; CARS; ADI-R; QPGS (Rome III); anthropometric measures	ADI-R; CARS; SSP	NA	CARS ↓ post-supplementation vs baseline; GSS ↓ post-supplementation; Bifidobacterium spp. ↑ post-supplementation; Lactobacillus spp. ↑ post-supplementation; Diet: NA	Probiotic supplementation (3 months); outcomes: CARS ↓; GSS ↓; Bifidobacterium spp. ↑; Lactobacillus spp. ↑; BMI ↓	Probiotic supplementation increased beneficial gut bacteria and reduced ASD symptom severity
Nalbant et al. (2022)	74	C-CS	2.5-8	90 (ASD 60; CG 30)	DSM-5	BSC; serum zonulin (ELISA); CARS; GSS	CARS; KSADS-PL	NR	Serum zonulin ↑ ASD vs CG; Serum zonulin–SSR (CARS): ↑; Serum zonulin–GSS: Ø; Diet: NA	NA	Higher serum zonulin levels associated with greater ASD symptom severity
Nirmalkar et al. (2022)	11	INT-LONG (second analysis Kang 2017)	7-16	38 (ASD 18; CG 20)	NR	FSC; shotgun metagenomic GS; BTA; GMC; functional pathway analysis (MetaCyc); alpha/beta diversity; correlation analyses; MTT protocol	CARS; ABC; SRS	PGI-III	Baseline GMC altered ASD vs CG; Microbial diversity ↑ post-MTT; Sulfur/oxidative stress pathways ↑ post-MTT; GSS ↓ post-MTT; SSR ↓ post-MTT; GSS–SSR: ↑; Diet: monitored	MTT (10 weeks); outcomes: GMC ↑; functional pathways ↑; GSS ↓; SSR ↓; sustained effects at 2-year follow-up	Microbiota Transfer Therapy induced long-lasting changes in gut microbiome composition and function in children with ASD, including restoration of sulfur metabolism and oxidative stress pathways; these microbial changes were accompanied by sustained improvements in gastrointestinal and core ASD symptoms, supporting a clinically relevant role of the microbiota–gut–brain axis.
Sherman et al. (2022)	85	RCT - secondary analysis	3-10	35 ASD (Probiotic 18; Placebo 17)	NR	RCT; probiotic supplementation (Lactobacillus plantarum PS128); FSC; 16S rRNA GS; BTA; GMC; GSS; serum inflammatory markers; oxytocin (ELISA); correlation analyses; LEfSe	ABC-2;SRS;CGI	NA	SpCO–SSR (SRS): ↑; Baseline GMC alpha diversity–SSR (SRS): ↓; GMC diversity changes–SSR: ↑ (probiotic group); GMC diversity changes–GSS: ↑ (probiotic group); Diet: NA	Probiotic supplementation (16 weeks); outcomes: GMC changes; SSR ↓; GSS ↓; no serious AEs	SpCO levels correlated with ASD symptom severity; probiotic treatment associated with coordinated changes in microbiota, inflammatory markers, and GI symptoms
Stewart et al. (2022)	87	PILOT INT-OL	12-17	30 ASD (Active treatment 20; placebo 10)	NR	Clinical trial; oral AB-2004 administration; BSC; urinary and plasma metabolomics (LC–MS/MS); GSS; GSRS; correlation analyses	ADOS-2; ABC; SRS-2; RBS-R;	PARS; CGI-I; CGI-S	Urinary/plasma gut-derived metabolites ↓ post-treatment vs baseline; GSS ↓ post-treatment; SSR (ABC-I, SRS-2) ↓ post-treatment; Diet: NA	AB-2004 treatment; outcomes: gut-derived metabolites ↓; GSS ↓; SSR ↓	AB-2004 treatment associated with reduced gut-derived microbial metabolites and improvements in anxiety, irritability, and GI symptoms
Turriziani et al. (2022)	91	INT-OL-PP	2-8	21 ASD T0/T1; n 17 ASD T2	DSM-5	PEG administration; BSC; urinary p-cresol (HPLC); Bristol Stool Scale; CARS; ADOS-2; SRS; RBS-R; CPRS	CARS; ADOS-2; RBS-R; SRS	CPRS	Stool consistency ↑ over time; SSR ↓ post-treatment vs baseline; Urinary p-cresol–SSR: Ø; Diet: NA	PEG treatment; outcomes: stool consistency ↑; SSR ↓; urinary p-cresol: NSA	Gut mobilization improved GI function and behavioral symptoms in constipated children with ASD
Açıkel et al. (2023)	19	C-CS	3-12	83 (ASD 53; CG 30)	DSM-5	BSC; TMAO; LBP	KSADS-PL-5	NA	TMAO, LBP: no differences ASD vs controls; ASD severity assoc: NA; Diet: NA	NA	No significant differences in serum TMAO or LBP levels between ASD and controls

Citation (Author, year)	Reference	Study design	Age range	Sample size	DSM and/or ICD diagnostical features	Methodological approaches across studies	Autism-related measures (diagnosis, severity, and symptom domains)	Behavioural scale	Clinical associations involving ASD symptoms, gastrointestinal features, and diet	Interventional studies and clinical outcomes	Main findings
Chamtouri et al. (2023)	29	C-CS	4-10	74 (ASD 28; SIB 18; CG 28)	DSM-5	Clinical diagnosis (NR; ADOS-2; ADI-R); ASD severity (CARS); FSC; fecal metabolomics (UHPLC); free amino acids, ammonium, biogenic amines, GABA; age stratification (4–7 vs 8–10 y); LDA; Kruskal–Wallis; Dunn; ANOVA; Mann–Whitney tests	ADI-R; ADOS-2; CARS	NR	Fecal total and individual amino acids ↑ in ASD vs SIB/CG (especially 4–7 y); Fecal histidine ↓ assoc with SSR (CARS); Biogenic amines, GABA, ammonium: Ø; Diet: NA	NA	Altered fecal amino acid profiles in ASD vs SIB/CG, more pronounced at younger ages; fecal amino acid profiles discriminated ASD from SIB/CG at 4–7 y but not at 8–10 y; lower fecal histidine associated with greater ASD severity
Cuomo et al. (2023)	34	C-CS	2-4	15 (ASD 8; CG 7)	DSM-5	FSC; host fecal DNA methylation profiling; 16S rRNA GS; BTA; fecal calprotectin	NR	NR	GMC–host DNA methylation assoc; Methylation patterns assoc with intestinal inflammation (calprotectin); SSR: NA; Diet: NA	NA	Distinct fecal host DNA methylation signatures associated with gut dysbiosis and intestinal inflammation in ASD
Radwan et al. (2023)	81	CSER	4-17	6 ASD (NO CG)	DSM-5	BSC; oxidative stress markers; glutathione supplementation; SRS; ABC; CGI; adverse events monitoring	ABC; SRS; CGI-S	NR	SSR ↓ post-supplementation vs baseline; GSS: NA; Diet: NA	Oral glutathione supplementation; outcomes: SSR ↓; GSS: NA	Glutathione supplementation associated with reported improvement in ASD-related symptoms
Yitik et al. (2023)	94	C-CS	4-12	90 (ASD 30; Siblings 30; CG 30)	DSM-5	FSC; qPCR (bacterial taxa); fecal calprotectin (ELISA); BSC; serum LBP (ELISA); serum sCD14 (ELISA); dietary habit questionnaire; GSS	CARS	NR	GSS–SSR: ↑; GMC–SSR: Ø; Dietary selectivity ↑ ASD vs CG	NA	GI symptom severity correlated with ASD symptom severity; gut microbiota composition did not
Chen et al. (2024)	31	C-CS	NR	1365 (ASD 898; CG 467)	NR	Secondary bioinformatics analysis; public 16S rRNA GS datasets (NCBI SRA); BTA (Kraken2, Bracken, Greengenes); alpha-, beta-, gamma-diversity (Hill numbers); diversity–area relationship modeling (PL-DAR, PLEC-DAR); AKP-based beta diversity; Wilcoxon tests; randomization testing	NR	NR	MC heterogeneity ↑ in ASD vs CG (AKP-supported); Alpha-diversity: NSA (dataset-dependent); GI features: NA; Diet: NA	NA	Across reanalyzed datasets, ASD GMC showed greater heterogeneity than controls, with differences driven mainly by rare taxa; beta-diversity patterns supported the Anna Karenina principle in a substantial proportion of datasets; diversity–ASD relationships were non-monotonic and highly heterogeneous
De Sales et al. (2024)	36	C-CS	6-8	61 (ASD 30; CG 31)	DSM-5	FSC; 16S rRNA GS (V3–V4); BTA; predicted functional pathway analysis (PICRUSt2, KEGG); sex-stratified analyses	CRIDI-ASD	NR	Predicted microbial metabolic pathways explored vs SSR: NSA; GI features: NA; Diet: NA	NA	Differences in gut microbiota composition and predicted microbial metabolic pathways between ASD and controls
Jung et al. (2024)	49	C-CS	3-18	456 (ASD 249; CG 101; Siblings 106)	DSM-5	FSC; 16S rRNA GS; enterotype clustering; clinical scales (SRS, CARS, VABS); machine learning models	NR	SRS-2; ABC; CARS	Specific microbial taxa assoc with SSR; associations varied by enterotype; GI features: NA; Diet: NA	NA	Enterotype-specific gut microbiome profiles observed in ASD
Kurokawa et al. (2024)	63	C-CS	6-12	98 (ASD 18; ADHD 19; ASD+ADHD 20; Siblings 13; Unrelated control 28)	DSM-5	FSC; 16S rRNA GS; BTA; GMC; alpha diversity (Chao1, Shannon, Faith's PD); dietary questionnaire; GSS; ADOS-2; CARS	ADOS-2; CARS; ABC; AQ; SSP	Conners 3rd Edition	GMC alpha diversity ↓ ASD vs CG; Dietary diversity: NSA; GSS: NA	NA	Lower gut microbiome alpha diversity in ASD vs non-related controls; no differences in dietary diversity
Martínez-González et al. (2024)	72	C-CS	3-41	265 ASD (NO CG)	DSM-5	GSS; SCQ; PSRS; RBS-R; correlation analyses; regression analyses	SCQ ; RBS-R; PSRS	NR	GSS–ASD traits (SCQ): ↑; GSS–pain: ↑; GSS–sensory reactivity: ↑; GSS–repetitive behaviors (RBS-R): ↑; Diet: NA	NA	Greater GI symptom severity associated with higher ASD traits and repetitive behaviors
Petropoulos et al. (2024)	77	C-CS	4-12	20 (ASD 40; ADHD 40; CG 40)	DSM-5	Hair elemental analysis; salivary cortisol; functional GI symptom questionnaire; group comparisons	NR	CBCL	GSS ↑ ASD vs CG; Internalizing/externalizing problems ↑ ASD, ADHD vs CG; Diet: NA	NA	Higher burden of functional GI symptoms and altered hair and salivary biomarkers observed in ASD
Reeves et al. (2024)	83	C-CS	11-18	18 (ASD 9; CG 9)	NR	GI mucosal biopsy sampling; 16S rRNA GS; BTA; GMC; alpha/beta diversity; PERMANOVA; two-way ANOVA	NR	NR	Mucosa-associated GMC differed ASD vs CG; Alpha diversity: NSA; GSS: NA; Diet: NA	NA	Region- and sex-specific alterations in mucosa-associated gut microbiota observed in ASD
Su et al. (2024)	88	C-CS	1-13	1627 (ASD 711; CG 916)	DSM-5	FSC; shotgun metagenomic GS; fungal ITS GS; BTA; GMC; functional pathway analysis; machine-learning models	AQ	NR	GMC differed ASD vs CG (bacterial + fungal); Functional microbial pathways differed ASD vs CG; GSS: NA; Diet: NA	NA	Distinct bacterial and fungal gut microbiota profiles and altered functional pathways observed in ASD
Wan et al. (2024)	92	C-CS	3-6	124 (ASD 60; CG 64)	DSM-5	FSC; virus-like particle DNA extraction; shotgun metagenomic GS; BTA; GMC; virome analysis; bacterial–viral network analysis; dietary diversity assessment	SRS-2	NR	Gut virome richness ↓ ASD vs CG; Bacteriome–virome interactions altered ASD vs CG; GSS: NA; Diet: Ø	NA	Reduced gut virome richness and altered bacteriome–virome interactions observed in ASD
Bhusri et al. (2025)	27	C-CS	3-35	95 (ASD 62; CG 33)	NR	FSC; 16S rRNA gene sequencing; microbiota diversity analysis; demographic stratification by age and sex	ADOS	NR	Overall gut microbiota composition: NSA (ASD vs CG); Sex- and age-stratified analyses: GMC differed by sex and age within ASD; GI features: NA; Diet: NA	NA	Gut microbiota composition and taxa differed between ASD and controls only when stratified by sex and age; Fusobacteriota/Fusobacterium enriched in ASD; age- and sex-specific microbial signatures identified
Chen et al. (2025)	32	Pooled Re-analysis	NR	1365 (ASD 898; CG 467)	NR	Secondary bioinformatics reanalysis; public gut microbiome datasets (16S rRNA sequencing); taxonomic classification (Kraken2, Bracken, Greengenes); microbial co-occurrence network construction; Stiffness Network Analysis; comparison with PM2RA and Netmoss; alpha-, beta-, and network-level analyses; random permutation testing (1,000); random forest validation (ROC/AUC)	NR	NR	NA	NA	Reanalysis across eight ASD gut microbiome datasets showed altered microbial network structure and increased cooperativity in ASD compared with controls; a biomarker subgroup (Bacteroides plebeius, Sutterella, Lachnospira, Prevotella copri) was identified based on network stiffness parameters; microbial networks in ASD displayed higher P/N ratios and significant relationship alterations (PM score = 0.72), supporting a non-monotonic and heterogeneous reorganization of gut microbiome interactions in ASD.

Citation (Author, year)	Reference	Study design	Age range	Sample size	DSM and/or ICD diagnostical features	Methodological approaches across studies	Autism-related measures (diagnosis, severity, and symptom domains)	Behavioural scale	Clinical associations involving ASD symptoms, gastrointestinal features, and diet	Interventional studies and clinical outcomes	Main findings
Di Benedetto et al. (2025)	37	C-CS	5-17; parents adult	53 (ASD 17; SIBLINGS 9; PARENTS 27)	NR	Dietary pattern assessment (questionnaires); FSC; 16S rRNA GS; BTA	NR	CBCL	Dietary patterns assoc with behavioral challenges; GMC/Mycobiome–behavior: Ø; GI features: NA	NA	Behavioral challenges in ASD associated with dietary patterns rather than gut microbiota or mycobiome composition
Gaougaou et al. (2025)	41	INT-OL-PP	4–11	23 ASD (NO CG)	NR	Open-label probiotic supplementation (Bio-K+, 14 weeks); ATEC; GSI; sleep questionnaires; EEG; actigraphy; dietary monitoring	ATEC	SCQ; BRIEF; CSHQ	ATEC ↓ post-treatment; GSI ↓ post-treatment; sleep symptoms ↓; Diet monitored: NSA	Daily probiotic beverage for 14 weeks; high acceptability/compliance; no product-related AEs; partial wash-out effect	Probiotic supplementation associated with short-term improvements in ASD-related behaviors, GI symptoms, and sleep
Mahdi et al. (2025)	59	C-CS	NR (children)	170 mother–child pairs (prenatal exposure analyses); 132 children (postnatal exposure analyses)	NR	BSC; serum vitamin B12; vitamin D3; folate (ELISA); FSC; 16S rRNA GS; BTA; GMC	NR	NR	Serum vitamin B12 ↓ ASD vs CG; Vitamin D3: NSA; Folate: NSA; Actinobacteria ↑ ASD vs CG; Firmicutes ↓ ASD vs CG; Bifidobacteriaceae ↑ ASD vs CG; Ruminococcaceae ↑ ASD vs CG; Species richness ↓ ASD vs CG; GSS: NA; Diet: NA	NA	Lower serum vitamin B12 levels and altered gut microbiota profiles observed in ASD vs controls
Osredkar et al. (2025)	75	C-CS	2-17	232 (ASD 161; CG 71)	NR	BSC; urinary metabolomics (LC-MS/MS); IS; PCS; TMAO; ADMA; SDMA; stratification by age, sex, SSR; non-parametric analyses (FDR)	CARS	NR	IS, PCS, TMAO, ADMA, SDMA: NSA ASD vs CG; PCS proportion ↑ severe ASD vs mild/moderate (CARS); IS/PCS ratio ↓ with SSR (CARS); GSS: NA; Diet: NA	NA	Uremic toxin ratios shifted with increasing ASD symptom severity
Sonbol et al. (2025)	86	CC	4-12	106 (ASD 53; CG 53)	DSM-5	BSC; serum zonulin (ELISA); CARS	CARS; GARS; MINI-KID	NR	Serum zonulin ↑ ASD vs CG; Serum zonulin–SSR (CARS): ↑; GSS: NA; Diet: NA	NA	Elevated serum zonulin levels associated with increased ASD symptom severity
Tang et al. (2025)	89	C-CS	3-6	64 recruited (ASD 32; CG 32); n 55 sequenced (ASD 25; CG 30)	DSM-5	Oral sample collection; 16S rRNA GS; BTA; MC; alpha/beta diversity; ROC analysis	SRS-2	NR	Oral MC diversity ↓ ASD vs CG; Oral taxa differed ASD vs CG; Oral MC–GSS: Ø; Diet: NA	NA	Reduced oral microbiota diversity and distinct taxa observed in young children with ASD
Wawer et al. (2025)	93	C-CS	2-14	80 (ASD 40; CG 40)	NR	BSC; serum IgA; serum IgE; serum IgM; complete blood count	NR	NR	Serum IgE ↓ ASD vs CG; Serum IgM ↓ ASD vs CG; GI features: NR; Diet: NR	NA	Lower serum IgE and IgM levels observed in ASD vs controls
Yuan et al. (2025)	95	CC	NR (children)	22 (ASD 11; CG 11)	DSM-5	FSC; viral metagenomic GS; virome analysis; BTA; alpha diversity (Shannon); beta diversity (PCA, unweighted UniFrac); phylogenetic analysis	NR	NR	Gut virome alpha diversity ↓ ASD vs CG; Gut virome beta diversity differed ASD vs CG; Microviridae ↓ ASD vs CG; Virome–SSR: Ø; Diet: NA	NA	Reduced gut virome alpha diversity and altered viral community composition observed in ASD
Zhong et al. (2025)	99	INT-OL-PP	NR (children)	101 ASD (NO CG)	DSM-5	FSC; oral sample collection (tongue coating); 16S rRNA GS (V3–V4); ASV inference (DADA2); BTA; GMC; alpha/beta diversity; LEfSe; correlation network analysis; functional prediction (FAPROTAX); WMT; longitudinal analyses; random forest; ROC analysis	ABC; CARS	NR	Tongue-coating MC differed ASD vs CG; Tongue-coating taxa–SSR (CARS/ABC): ↑; Tongue-coating taxa–sleep (SDSC): ↑; Tongue–gut taxa correlations: ↑; Post-WMT: SSR ↓; Post-WMT: GSS: NA; Diet: partially standardized (Ø as outcome)	WMT (multiple courses); outcomes: SSR ↓ (CARS, ABC); sleep disturbance ↓ (SDSC); GMC/MC shifts; mild AEs only; no serious AEs	Children with ASD exhibit distinct tongue-coating microbiota profiles closely linked to gut microbiota and clinical symptom severity; WMT improves core ASD symptoms and sleep while reshaping both gut and tongue-coating microbiota; pre-treatment tongue-coating microbiota combined with clinical features can predict WMT efficacy with accuracy comparable to gut microbiota–based models, supporting the relevance of an oral–gut–brain axis in ASD.
Zhu et al. (2025)	101	CS	NR (children)	Genotyping cohort: 1255 (ASD 610; CG 645); Gut microbiota sub-cohort n 158 (ASD 87; CG 71)	DSM-5	SNP genotyping (lncRNA variants, MassARRAY); FSC; 16S rRNA GS; BTA; GMC; correlation and mediation analyses	NR	NR	GMC differed ASD vs CG; lncRNA SNPs–ASD risk: ↑/↓ (variant-specific); SNPs–GMC: assoc; GMC–ASD risk mediation: Ø; GI features: NA; Diet: NA	NA	Specific functional lncRNA variants were associated with ASD risk; gut microbiota composition differed in ASD and was associated with lncRNA variants but did not mediate genetic risk

ABBREVIATION: **↑** increase/higher; **↓** decrease/lower; **≠** different/altered; **Ø** no association; **NSA** non-significant (statistically); **NA** not assessed; **NR** not reported; **ASD** autism spectrum disorder; **ADHD** attention-deficit/hyperactivity disorder; **CG** control group; **TD** typically developing; **SIB** siblings; **GI** gastrointestinal; **GSS** gastrointestinal symptoms (generic); **GSI** Gastrointestinal Severity Index; **GSRS** Gastrointestinal Symptom Rating Scale; **ADOS** Autism Diagnostic Observation Schedule; **ADOS-2** Autism Diagnostic Observation Schedule, Second Edition; **ADI-R** Autism Diagnostic Interview–Revised; **CARS** Childhood Autism Rating Scale; **SRS** Social Responsiveness Scale; **SRS-2** Social Responsiveness Scale, Second Edition; **SCQ** Social Communication Questionnaire; **ABC** Aberrant Behavior Checklist; **ATEC** Autism Treatment Evaluation Checklist; **RBS-R** Repetitive Behavior Scale–Revised; **SSP** Short Sensory Profile; **AQ** Autism Spectrum Quotient; **PARS** Pediatric Anxiety Rating Scale; **K-SADS-PL** Kiddie Schedule for Affective Disorders and Schizophrenia; **BRIEF** Behavior Rating Inventory of Executive Function; **CBCL 6–18** Child Behavior Checklist, Ages 6–18; **TRF** Teacher Report Form; **CGI-S** Clinical Global Impressions – Severity; **CGI-I** Clinical Global Impressions – Improvement; **ADHD-RS-IV** Attention-Deficit/Hyperactivity Disorder Rating Scale IV; **SCFA** short-chain fatty acids; **TMAO** trimethylamine N-oxide; **LBP** lipopolysaccharide-binding protein; **TPP** thiamine pyrophosphate; **TMP** thiamine monophosphate; **PCS** p-cresyl sulfate; **IS** indoxyl sulfate; **HPHPA** 3-(3-hydroxyphenyl)-3-hydroxypropionic acid; **I-FABP** intestinal fatty acid–binding protein; **IL-6** interleukin-6; **TNF-α** tumor necrosis factor alpha; **TGF-β** transforming growth factor beta; **S100B S100** calcium-binding protein B; **NSE** neuron-specific enolase; **GMC** gut microbiota composition; **OTU** operational taxonomic unit; **ASV** amplicon sequence variant; **ARG** antibiotic resistance genes; **16S rRNA 16S** ribosomal RNA gene sequencing; **ITS** internal transcribed spacer sequencing; **qPCR** quantitative polymerase chain reaction; **UHPLC** ultra-high-performance liquid chromatography; **LC-MS/MS** liquid chromatography–tandem mass spectrometry; **GC-MS** gas chromatography–mass spectrometry; **1H-NMR** proton nuclear magnetic resonance spectroscopy; **BTA** bioinformatic taxonomic analysis; **QIIME2** microbiome bioinformatics pipeline; **DADA2** Divisive Amplicon Denoising Algorithm 2; **Kraken2** taxonomic classifier; **Bracken** abundance estimation tool; **PICRUSt2** Phylogenetic Investigation of Communities by Reconstruction of Unobserved States 2; **KEGG** Kyoto Encyclopedia of Genes and Genomes; **AKP** Anna Karenina principle (microbiome heterogeneity); **PL-DAR** power-law diversity–area relationship; **PLEC-DAR** power-law with exponential cutoff diversity–area relationship; **PM2RA** Profile Monitoring for Microbial Relationship Alteration; **SNA** stiffness network analysis; **FMT** fecal microbiota transplantation; **MTT** Microbiota Transfer Therapy; **PEG** polyethylene glycol; **AB-2004** gastrointestinal-restricted adsorbent; **CS** cross-sectional study; **CC** case–control study; **C-CS** comparative cross-sectional study; **COH** cohort study; **COH-P** prospective cohort study; **RCT** randomized controlled trial; I**NT-OL** open-label interventional study; **INT-OL-PP** open-label pre–post interventional study; **INT-LONG** longitudinal interventional study; **CSER** case series; **SSR** autism symptom severity rating.
