# Supplementary material for: The microbiota–gut–brain axis as a modulator of symptom expression in autism spectrum disorder, with exploratory insights into ADHD: evidence from a structured narrative review on paediatric population
Source: Front Child Adolesc Psychiatry. 2026 May 28;5:1835043. doi: 10.3389/frcha.2026.1835043 (PMC13253956; doi:10.3389/frcha.2026.1835043)
Supplement: Supplementary file 2 [file Table2.pdf]

Citation (Author, year)	Study design	Domain 1: Risk of bias arising from the randomization process	Domain 2: Risk of bias due to deviations from the intended interventions	Domain 3: Missing outcome data	Domain 4: Risk of bias in measurement of the outcome	Domain 5: Risk of bias in selection of the reported result	Overall risk of bias
Kumperscak et al. (2020)	Pilot RCT	Randomization reported, but sequence generation/allocation concealment not fully described SOME CONCERNS	Double-blind design, supervised administration, high adherence LOW RISK	Three randomized participants excluded before baseline SOME CONCERNS	Main outcomes include subjective reports despite blinding SOME CONCERNS	No prespecified primary outcome or trial registry identifier reported SOME CONCERNS	SOME CONCERNS
Elhossiny et al. (2023)	RCT	Computer-generated randomization LOW RISK	Open-label design with no placebo control SOME CONCERNS	Four post-randomization losses in probiotic arm SOME CONCERNS	Assessor-blinded, but parent-rated outcomes in open-label trial SOME CONCERNS	Registered trial with prespecified primary and secondary outcomes LOW RISK	SOME CONCERNS
Lawrence et al. (2025)	RCT	Randomization reported, but sequence generation/allocation concealment not fully described SOME CONCERNS	Double-blind placebo-controlled intervention LOW RISK	High and unbalanced attrition (27/80, 33.75%; 18 vs 9). Mixed model ANCOVA reported, but manuscript also states withdrawn participants were omitted from analysis; handling of missing data remains unclear. SOME CONCERNS	Blinded trial, but primary outcome includes parent/teacher ratings SOME CONCERNS	Prospectively registered trial with prespecified outcomes LOW RISK	SOME CONCERNS

Supplementary Table 3a. Methodological appraisal of unique randomized controlled trials (RCTs) included in the review, performed using the Cochrane Risk of Bias 2 (RoB 2) framework.
