# Supplementary material for: The microbiota–gut–brain axis as a modulator of symptom expression in autism spectrum disorder, with exploratory insights into ADHD: evidence from a structured narrative review on paediatric population
Source: Front Child Adolesc Psychiatry. 2026 May 28;5:1835043. doi: 10.3389/frcha.2026.1835043 (PMC13253956; doi:10.3389/frcha.2026.1835043)
Supplement: Supplementary file 3 [file Table3.pdf]

Citation (Author, year)	Study design	Parent study	Domain 1: Risk of bias arising from the randomization process	Domain 2: Risk of bias due to deviations from the intended interventions	Domain 3: Missing outcome data	Domain 4: Risk of bias in measurement of the outcome	Domain 5: Risk of bias in selection of the reported result	Overall risk of bias
Sherman et al. (2022)	RCT - secondary analysis	Kong XJ et al., (2021)	Randomized 1:1 ratio, CONSORT guidelines followed<br>LOW RISK	Double-blind, placebo-controlled<br>LOW RISK	High attrition rate (from 35 to 27 subjects).<br>SOME CONCERNS	Mixed subjective and newly added biomarker outcomes in secondary analysis<br>SOME CONCERNS	Post-hoc exploratory analysis. Outcomes were not pre-specified in the original trial.<br>HIGH RISK	HIGH RISK
Billeci et al. (2022)	RCT - secondary analysis	Santocchi et al. (2016)	Randomized 1:1 ratio<br>LOW RISK	Double-blind, placebo-controlled<br>LOW RISK	Check for EEG technical drop-outs<br>SOME CONCERNS	Objective EEG instrumentation.<br>LOW RISK	Secondary outcome analysis.<br>SOME CONCERNS	SOME CONCERNS
Pärtty et al. (2015)	RCT - secondary analysis	Secondary long-term follow-up analysis of a parent RCT	Randomization reported, but sequence generation/allocation concealment not fully described<br>SOME CONCERNS	Double-blind, placebo-controlled<br>LOW RISK	Substantial attrition at 13-year follow-up (75/159 analyzed)<br>HIGH RISK	ICD-10 standardized criteria<br>LOW RISK	Secondary outcome analysis.<br>SOME CONCERNS	SOME CONCERNS
Stevens et al. (2019)	RCT - secondary analysis	Rucklidge et al. (2018)	Randomization reported, but sequence generation/allocation concealment not fully described<br>SOME CONCERNS	Double-blind design, supervised administration, high adherence<br>LOW RISK	One post-randomization exclusion in placebo arm (antibiotic exposure)<br>SOME CONCERNS	Objective microbiome outcomes assessed with standardized sequencing methods<br>LOW RISK	Exploratory microbiome substudy not prespecified at trial initiation<br>HIGH RISK	HIGH RISK
Robinette et al. (2024)	RCT - secondary analysis	Johnstone et al., (2022)	Double-blind placebo-controlled RCT with 3:2 randomization<br>LOW RISK	Blinded intervention with identical placebo capsules<br>LOW RISK	Modified ITT subset with LOCF for missing week 8 CGI-I<br>SOME CONCERNS	Blinded clinician-rated CGI-I outcome assessment<br>LOW RISK	Secondary moderator analysis with exploratory multiple comparisons<br>SOME CONCERNS	SOME CONCERNS

Citation (Author, year)	Study design	Parent study	Domain 1: Risk of bias arising from the randomization process	Domain 2: Risk of bias due to deviations from the intended interventions	Domain 3: Missing outcome data	Domain 4: Risk of bias in measurement of the outcome	Domain 5: Risk of bias in selection of the reported result	Overall risk of bias
Ast (2025)	RCT-secondary analysis	Johnstone et al., (2022)	Double-blind randomized parent RCT<br>LOW RISK	Blinded intervention with identical placebo LOW RISK	Selected microbiome subgroup with post-randomization exclusions<br>HIGH RISK	Objective microbiome outcome assessed with standardized sequencing<br>LOW RISK	Exploratory subgroup analysis with non-corrected multiple testing<br>HIGH RISK	HIGH RISK

Supplementary Table 3b. Methodological appraisal of secondary analyses derived from previously reported randomized controlled trial (RCT) datasets, performed using the Cochrane Risk of Bias 2 (RoB 2) framework, with particular attention to selective outcome reporting and post hoc analytical decisions.
