# Supplementary material for: The microbiota–gut–brain axis as a modulator of symptom expression in autism spectrum disorder, with exploratory insights into ADHD: evidence from a structured narrative review on paediatric population
Source: Front Child Adolesc Psychiatry. 2026 May 28;5:1835043. doi: 10.3389/frcha.2026.1835043 (PMC13253956; doi:10.3389/frcha.2026.1835043)
Supplement: Supplementary file 4 [file Table4.pdf]

Citation (Author, year)	Study design	Parent study	MINORS 1: Clearly stated aim	MINORS 2: Inclusion of consecutive patients	MINORS 3: Prospective data collection	MINORS 4: Endpoints appropriate to the aim	MINORS 5: Unbiased assessment of endpoints	MINORS 6: Appropriate follow-up period	MINORS 7: Loss to follow-up <5%	MINORS 8: Prospective calculation of study size	Total MINORS score (/16)
Kang et al. (2017)	PILOT INT-OL-PP		Clearly stated interventional aim and predefined primary/secondary outcomes<br>2	Recruitment described with eligibility criteria, but not clearly consecutive<br>1	Prospective longitudinal pre-post data collection<br>2	Clinical and microbiome endpoints appropriate to study aim<br>2	Open-label design with non-blinded outcome assessment, despite standardized measures<br>1	Ten-week treatment plus 8-week follow-up appropriate to exploratory aims<br>2	No loss to follow-up was reported; all 18 ASD participants completed the 18-week study<br>2	No prospective sample size calculation<br>0	12/16
Liu et al. (2017)	PILOT INT-OL-PP		Clearly stated interventional aim and predefined outcomes<br>2	Recruitment and eligibility criteria described, but consecutive inclusion not clearly stated<br>1	Prospective pre-post data collection<br>2	Clinical and microbiome endpoints appropriate to study aim<br>2	Single-blind design reported, but blinded outcome assessment not clearly described; several outcomes based on parent-rated scales<br>1	Six-month follow-up appropriate to exploratory aims<br>2	Main cohort completed 6-month follow-up, with no relevant attrition for primary pre-post analysis<br>2	No prospective sample size calculation<br>0	12/16
Bent et al. (2018)	PILOT INT-OL-PP		Clearly stated interventional aim and predefined outcomes<br>2	Recruitment described and all school families invited, but no true consecutive clinical inclusion<br>1	Prospective pre-post open-label data collection with prospective trial registration<br>2	Clinical (ABC/SRS) and urinary metabolomic endpoints appropriate to the study aim<br>2	Open-label design with parent-rated outcomes and explicit risk of expectation bias<br>0	Twelve-week follow-up appropriate to exploratory aims<br>2	Substantial attrition from consented participants to completers (21 to 15; >5%)<br>0	A priori power justification for detecting clinically relevant metabolite-symptom correlations ($r \geq 0.6$) with n=15<br>2	11/16

Citation (Author, year)	Study design	Parent study	MINORS 1: Clearly stated aim	MINORS 2: Inclusion of consecutive patients	MINORS 3: Prospective data collection	MINORS 4: Endpoints appropriate to the aim	MINORS 5: Unbiased assessment of endpoints	MINORS 6: Appropriate follow-up period	MINORS 7: Loss to follow-up <5%	MINORS 8: Prospective calculation of study size	Total MINORS score (/16)
Kang et al. (2019)	INT-OL-LONG		Clearly stated long-term follow-up aim assessing safety and efficacy of MTT 2	Closed cohort follow-up including all 18 original participants, with complete participation 2	Prospective long-term follow-up data collection with repeat clinical and microbiota assessments 2	GI, autism-related, safety and microbiota endpoints appropriate to study aim 2	Open-label parent-rated assessments remain vulnerable to expectation/placebo effects, although CARS included professional evaluator rating 1	Two-year follow-up highly appropriate to long-term exploratory aims 2	No loss to follow-up at 2-year reassessment (18/18) 2	No prospective sample size calculation for the follow-up study 0	13/16
Meguid et al. (2022)	INT-OL-PP		Clearly stated exploratory aim evaluating probiotic supplementation as adjuvant therapy in ASD 2	Recruitment setting, eligibility and exclusions described, but consecutive inclusion not reported 1	Prospective pre-post data collection over 3 months 2	Clinical, gastrointestinal and microbiological endpoints appropriate to study aim 2	No blinded outcome assessment reported; several outcomes rely on parent report and clinician reassessment in an uncontrolled design 0	Three-month follow-up appropriate to exploratory probiotic intervention aims 2	Attrition/completion not clearly reported despite 40 enrolled participants, limiting certainty on loss to follow-up 1	No prospective sample size calculation reported 0	10/16
Nirmalkar et al. (2022)	INT-LONG (secondary analysis)	Kang et al. (2017)	Clearly stated mechanistic aim using shotgun metagenomics on previously collected MTT samples 2	Closed predefined cohort derived from the original ASD trial (18 participants; 16 with 2-year fecal samples) 2	Longitudinal samples were originally prospectively collected, but shotgun metagenomic analysis was performed secondarily on previously extracted fecal DNA 1	Taxonomic and functional metagenomic endpoints appropriate to the mechanistic study aim 2	Primary outcomes were objective laboratory-based sequencing and bioinformatic measures, limiting observer bias despite open-label parent trial context 2	Ten-week and two-year timepoints appropriate for short- and long-term microbiome functional follow-up 2	Two-year fecal sampling incomplete (16/18), exceeding 5% loss for the key long-term analysis 0	No prospective sample size calculation for the shotgun metagenomic analysis; small sample acknowledged 0	11/16

Citation (Author, year)	Study design	Parent study	MINORS 1: Clearly stated aim	MINORS 2: Inclusion of consecutive patients	MINORS 3: Prospective data collection	MINORS 4: Endpoints appropriate to the aim	MINORS 5: Unbiased assessment of endpoints	MINORS 6: Appropriate follow-up period	MINORS 7: Loss to follow-up <5%	MINORS 8: Prospective calculation of study size	Total MINORS score (/16)
Stewart et al. (2022)	PILOT INT-OL		Clearly stated phase 1b/2a aim with predefined primary (safety/tolerability), secondary (target engagement) and exploratory behavioral endpoints 2	Screening, eligibility and multicenter recruitment described, but consecutive inclusion not reported 1	Prospective registered open-label interventional data collection with scheduled baseline, end-of-treatment and follow-up assessments 2	Safety, laboratory, metabolite target-engagement, GI and behavioral endpoints appropriate to study aims 2	Open-label design introduces risk of bias for subjective outcomes, although primary/secondary endpoints include objective laboratory and metabolite measures 1	Eight-week treatment plus four-week follow-up appropriate to early-phase safety and target-engagement aims 2	Attrition exceeded 5% from enrollment to final visit (30 enrolled, 24 completed final visit) 0	No formal prospective sample size calculation reported 0	10/16
Turriziani et al. (2022)	INT-OL-PP		Clearly stated prospective aim evaluating behavioral, gastrointestinal and urinary p-cresol changes after gut mobilization with PEG in chronically constipated autistic children 2	Recruitment setting and detailed eligibility criteria described, but consecutive inclusion not reported 1	Prospective registered open-label longitudinal data collection at baseline, 1 month and 6 months 2	Behavioral, stool consistency and urinary p-cresol endpoints appropriate to study aim, with concomitant treatments kept stable 2	Open-label design with non-blinded parent-reported measures and non-blinded clinician ratings, explicitly acknowledged by authors as a limitation 0	Six-month follow-up with interim 1-month assessment appropriate to exploratory gut-mobilization aims 2	Attrition exceeded 5% (25 initially recruited, 21 assessed at T0/T1, 17 at T2), despite drop-outs being attributed to COVID-19 0	No prospective sample size calculation reported; reported power/effect size analyses do not constitute prospective planning 0	9/16

Citation (Author, year)	Study design	Parent study	MINORS 1: Clearly stated aim	MINORS 2: Inclusion of consecutive patients	MINORS 3: Prospective data collection	MINORS 4: Endpoints appropriate to the aim	MINORS 5: Unbiased assessment of endpoints	MINORS 6: Appropriate follow-up period	MINORS 7: Loss to follow-up <5%	MINORS 8: Prospective calculation of study size	Total MINORS score (/16)
Gaougaou et al. (2025)	INT-OL-PP		Clearly stated primary aims assessing probiotic acceptability, safety and protocol feasibility, with only preliminary exploratory evaluation of behavioral, GI and sleep outcomes 2	Recruitment strategy and eligibility criteria clearly described, but inclusion was not consecutive and relied on social media response plus chart-based recruitment 1	Prospective open-label non-randomized longitudinal data collection over 30 weeks with five predefined time-points 2	Acceptability, adverse-event, compliance, retention and data-completion endpoints were highly appropriate to the safety/feasibility aims, with exploratory behavioral/GI/sleep measures clearly secondary 2	Open-label design limits objectivity of exploratory symptom outcomes, but primary feasibility/safety endpoints were largely procedural and less susceptible to observer bias 1	Study duration including pre-treatment stabilization, 14-week intervention and 8-week washout was appropriate to safety, feasibility and persistence aims 2	Attrition was below 5% (23 recruited, 1 post-T-8pre withdrawal, 22 completed), 2	No prospective sample size calculation was performed 0	12/16
Zhong et al. (2025)	INT-OL-PP retrospective WMT cohort analysis		Clearly stated aim investigating tongue-coating microbiota in ASD, its association with symptoms/gut microbiota, and its predictive value for WMT response 2	Children admitted for WMT within a defined period were eligible, but consecutive inclusion was not explicitly reported and patients without follow-up were excluded 1	Retrospective cohort design; data were extracted from medical records rather than prospectively collected for this analysis 0	Clinical scales (CARS, ABC, SDSC) and paired tongue/fecal microbiota measures were appropriate to the biomarker and response-prediction aims 2	Endpoint assessment was partially objective but not blinded; CARS was clinician-administered, whereas ABC/SDSC were parent-reported and response categories were defined post-hoc 1	Repeated WMT courses with serial pre/post assessments across treatment cycles provided an appropriate observation period for exploratory response tracking 2	Attrition/loss to follow-up could not be reliably determined because patients lacking follow-up data were excluded a priori and reporting across the 51-child vs 40-child analytic samples was not fully transparent 0	No prospective sample size calculation reported 0	8/16

Citation (Author, year)	Study design	Parent study	MINORS 1: Clearly stated aim	MINORS 2: Inclusion of consecutive patients	MINORS 3: Prospective data collection	MINORS 4: Endpoints appropriate to the aim	MINORS 5: Unbiased assessment of endpoints	MINORS 6: Appropriate follow-up period	MINORS 7: Loss to follow-up <5%	MINORS 8: Prospective calculation of study size	Total MINORS score (/16)
Niu et al. (2025)	INT-OL single arm		Clearly stated prospective aim to evaluate the safety and exploratory efficacy of a probiotic mixture in children with ADHD 2	Eligibility criteria and recruitment flow were clearly described, but consecutive enrollment was not explicitly reported 1	Prospective open-label single-arm clinical trial with predefined assessments at baseline, week 5, and week 9 and registered protocol 2	Primary and secondary endpoints were appropriate to the study aim, including BRIEF-II, SNAP-IV, adverse events, and biochemical safety monitoring 2	Outcome assessment relied on standardized but caregiver-reported scales in a non-blinded open-label design, increasing risk of expectancy and reporting bias 1	Eight-week intervention with interim and end-of-treatment assessments was appropriate for exploratory short-term efficacy and safety 2	Attrition exceeded 5% (8/50 participants withdrew; final n=42), 0	A prospective sample size calculation was reported based on the BRIEF-II GEC primary endpoint, including dropout adjustment 2	13/16

Supplementary Table 4. Methodological appraisal of non-randomized open-label interventional studies included in the review, evaluated using the Methodological Index for Non-Randomized Studies (MINORS) criteria for non-comparative designs, with item-level scores and total score (/16).
